# Supplementary material for: Elasto-magnetic instabilities for amplified actuation and mechanical memory
Source: Nat Commun. 2026 Jan 10;17:1511. doi: 10.1038/s41467-025-68225-y (PMC12890967; doi:10.1038/s41467-025-68225-y)
Supplement: Supplementary file 1 — Supplementary Information [file 41467_2025_68225_MOESM1_ESM.pdf]

Supplementary Information for

Elasto-magnetic instabilities for amplified actuation and mechanical memory

Seong-Yu Choi[1]†, Ji-Sung Park[2,3]†, Won Jun Song[1], Maga Kim[1], Yun Hyeok Lee[1], Yong Eun Cho[1], Hakjun Lee[1], Ho-Young Kim[2,4*], Jeong-Yun Sun[1,5*]

[1]Departmant of Materials Science and Engineering, Seoul National University, Seoul 08826, Republic of Korea

[2]Departmant of Mechanical Engineering, Seoul National University, Seoul 08826, Republic of Korea

[3]Institute of Mechanical Engineering, École Polytechnique Fédérale de Lausanne, 1015 Lausanne, Switzerland

[4]Institute of Advanced Machines and Design, Seoul National University, Seoul 08826, Republic of Korea

[5]Research Institute of Advanced Materials (RIAM), Seoul National University, Seoul 08826, Republic of Korea

† These authors contributed equally to this work.

Correspondence to: hyk@snu.ac.kr, jysun@snu.ac.kr

This PDF file includes:

Supplementary Note 1 to 6

Supplementary Table 1 to 2

Supplementary Figure 1 to 29

Other Supplementary Materials for this manuscript include the following:

Supplementary Videos 1 to 7

Table of contents

Supplementary Note 1 & Table 1. Analysis of forces acting on a magnet 4
Supplementary Note 2. Static force analysis of system. 8
Supplementary Note 3. Resonance frequency in our system 9
Supplementary Note 4. Design map for optimal region 11
Supplementary Table 2. Characteristic parameters and forces for four cases 12
Supplementary Note 5. Characterization of C-EsMV based on the initial position of membrane 13
Supplementary Note 6. Efficiency of this system. 15
Supplementary Figure 1. Schematic representation of potential energy in attractively coupled magnets, an elastic membrane, and an electromagnet 16
Supplementary Figure 2. Static force analysis of non-coupled (NC-EsMV) and coupled (C-EsMV) systems 17
Supplementary Figure 3. Static analysis of equilibrium and energy as current is varied. 18
Supplementary Figure 4. Energy conversion efficiency and cyclic stability of vibrational hysteresis. 19
Supplementary Figure 5. Numerical results for magnet's position (z), net force (F), and potential and kinetic energies during a single actuation cycle of NC-EsMV and C-EsMV. 20
Supplementary Figure 6. Structural parameters and magnetic scalar potential in the farthest and closest positions of the magnet from the electromagnet phases 21
Supplementary Figure 7. Design map for different cases with varying elastic and magnetic forces 22
Supplementary Figure 8. Comparison of thermal stability for each system 23
Supplementary Figure 9. Characterization of C-EsMV based on the initial position (*D*) 24
Supplementary Figure 10. Comparison of four different C-EsMV systems with varied current and membrane modulus conditions 25
Supplementary Figure 11. Initial position-amplitude plot of C-EsMV with varying conditions of elastic membrane, but with identical characteristic membrane force F^{*}_{ES}. 26
Supplementary Figure 12. Experimental verification of vibrational hysteresis in C-EsMV. 27
Supplementary Figure 13. Numerical results of electrical shooting behavior and motion hysteresis in C-EsMV 28
Supplementary Figure 14. Stability and efficient energy conversion after shooting. 29
Supplementary Figure 15. Change in amplifiable regions based on electromagnet input current 30
Supplementary Figure 16. Vibration behavior in a C-EsMV system at different frequencies. 31
Supplementary Figure 17. Time-resolved dynamic analysis of membrane motion under resonance at different dynamic viscosities.. 32
Supplementary Figure 18. Vibration behavior in a C-EsMV system with different waveforms. 33
Supplementary Figure 19. Experimental setup for impact force measurement and real-time peak force. 34
Supplementary Figure 20. The effect of anisotropy on amplified vibration 35
Supplementary Figure 21. Structure and working mechanism for flapping wing demonstration. 36
Supplementary Figure 22. Numerical verification of mechanically triggered amplification. 37
Supplementary Figure 23. Mechanism of vibrational hysteretic behavior in mechanical memory 38
Supplementary Figure 24. Dynamic vibration response of the mechanical memory in standby and memorized states. 39
Supplementary Figure 25. Experimental setup for mechanical memory and its operating modes. 40

Supplementary Figure 26. Trigger force characterization with elastic spacers of different elastic moduli. 41
Supplementary Figure 27. Effect of membrane damping on mechanical memory 42
Supplementary Figure 28. The entire process of non-volatile mechanical memorization. 43
Supplementary Figure 29. Non-volatile mechanical memorization by directly pressing the magnet. 44
Supplementary References 45

Supplementary Note 1. Analysis of forces acting on a magnet

We here provide details of analytical force models given in main text. We first observe a case when a membrane of Young's modulus E, Poisson's ratio ν, radius R_m, prestretch λ_p, and final thickness h_m with a magnet of radius R_a attached on the center is vertically displaced by amplitude, Δ, as depicted in Supplementary Fig. 4a. Due to axisymmetry, lateral force components cancel out, and the net force acts purely in the vertical direction. As the magnet restrains the stretching of the middle region, the stretched membrane can be modeled as a side surface of a truncated cone which allows us to express radial and circumferential stretches λ_r and λ_θ as,

$$\lambda_r = \lambda_p \lambda_{1,v} = \lambda_p \frac{\sqrt{(R_m - R_a)^2 + \Delta^2}}{R_m - R_a}, \qquad (1)$$

$$\lambda_\theta = \lambda_p \lambda_{2,v} = \lambda_p$$

Here, $\lambda_{1,v}$ and $\lambda_{2,v}$ represent the additional radial and circumferential stretches, respectively, due to vertical displacement Δ, and are scaled by the initial prestretch λ_p.

Therefore, we can calculate the total strain potential energy of the membrane $E_{ES} = A_{ES} \frac{E}{4(1+\nu)} (\lambda_r^2 + \lambda_\theta^2 + \lambda_r^{-2} \lambda_\theta^{-2} - 3)$ based on the neo-Hookean incompressible model[1], where A_{ES} is the membrane area. Substituting Eq. (1) into this relation yields:

$$E_{ES} = \frac{\pi E}{4(1+\nu)} h_m \lambda_p^2 (R_m^2 - R_a^2) \left[\frac{(R_m - R_a)^2 + \Delta^2}{(R_m - R_a)^2} + 1 + \frac{1}{\lambda_p^6} \frac{(R_m - R_a)^2}{(R_m - R_a)^2 + \Delta^2} - \frac{3}{\lambda_p^2} \right]. \qquad (2)$$

Differentiating E_{ES} by $z = D - \Delta$, we get the elastic membrane force F_{ES} as,

$$F_{ES} = -\frac{\partial E_{ES}}{\partial z} = \frac{\pi E}{2(1+\nu)} h_m \lambda_p^2 (R_m^2 - R_a^2) \left[\frac{1}{(R_m - R_a)^2} - \frac{1}{\lambda_p^6} \frac{(R_m - R_a)^2}{((R_m - R_a)^2 + (D - z)^2)^2} \right] (D - z), \qquad (3)$$

where D is the initial spacing between the electromagnet and the magnet. By neglecting the higher order term and rearranging, the elastic force F_{ES} can be approximated as:

$$F_{ES} = \frac{\pi E}{2(1+\nu)} h_m \lambda_p^2 (R_m + R_a) \frac{D - z}{R_m - R_a} = F_{ES}^* \frac{D - z}{R_m - R_a}, \qquad (4)$$

where F^{*}_{ES} corresponds to the magnitude of the elastic force when the membrane achieves a radial stretch $\lambda_r = \sqrt{2}$, when inclination angle (of the isosceles trapezoid formed by the membrane) reaches 45°.

The damping force F_d of the membrane is also considered. We use the Kelvin-Voigt model comprising a spring and a dashpot in parallel to describe the viscous damping effect of membrane during vibration[2].

$$F_\mathrm{d} = \eta \frac{\dot{\lambda}_1}{\lambda_1} = -\eta\pi h_\mathrm{m}(R_\mathrm{m}^2 - R_\mathrm{a}^2)\frac{(D-z)^2}{[(R_\mathrm{m}-R_\mathrm{a})^2 + (D-z)^2]^2}\dot{z} \tag{5}$$

where η is the viscosity, and the dot notations in $\dot{\lambda}_1$ and $\dot{z}$ denote time derivatives.

The magnet-magnet attraction force F_MM can be obtained by the Bio-Savart law, which is written as[3,4]

$$F_\mathrm{MM} = \frac{\mu_0}{2} M_\mathrm{a} M_\mathrm{b} R_\mathrm{a} R_\mathrm{b} \int_0^{2\pi} \cos\delta\, d\delta \int_{-\frac{h_\mathrm{a}}{2}}^{\frac{h_\mathrm{a}}{2}} \left[\frac{1}{\sqrt{R_\mathrm{a}^2 + R_\mathrm{b}^2 - 2R_\mathrm{a}R_\mathrm{b}\cos\delta + \left(\frac{h_\mathrm{a}}{2} + 2z + h - z_\mathrm{a}\right)^2}} - \frac{1}{\sqrt{R_\mathrm{a}^2 + R_\mathrm{b}^2 - 2R_\mathrm{a}R_\mathrm{b}\cos\delta + \left(\frac{h_\mathrm{a}}{2} + 2z + h + h_\mathrm{b} - z_\mathrm{a}\right)^2}} \right] dz_\mathrm{a} \tag{6}$$

where μ_0 is the vacuum permeability, M_a and M_b are the magnetizations, R_a and R_b are the radii, h_a and h_b are the thicknesses of the magnets placed on opposite side of electromagnet, and h is the thickness of the electromagnet. To simplify notation, we group the geometric contributions into a single term $\Omega \sim f(R_\mathrm{a}, R_\mathrm{b}, h_\mathrm{a}, h_\mathrm{b}, h, z)$, and express the force compactly as: $F_\mathrm{MM} = \frac{\mu_0}{2} M_\mathrm{a} M_\mathrm{b} \Omega$.

Characteristic value $F^{*}_\mathrm{MM} = \frac{\mu_0}{2} M_\mathrm{a} M_\mathrm{b} \Omega|_{z=0}$ is numerically determined by evaluating Eq. (6) when the magnets are positioned at the bottom surface of the electromagnet to standardize the magnet strength of varying geometries.

The electromagnetic force F_EM between the electromagnet and the magnet on one side (denoted by subscript a for clarity) is given by the summation of those from circular current loop in radial and longitudinal directions[3,4]:

$$F_{\mathrm{EM}} = \frac{\mu_0}{2} M_{\mathrm{a}} R_{\mathrm{a}} I \sum_{j=1}^{n_{\mathrm{s}}} \sum_{i=1}^{n_{\mathrm{t}}} \int_0^{2\pi} r_i \cos\delta \left[\frac{1}{\sqrt{R_{\mathrm{a}}^2 + r_i^2 - 2R_{\mathrm{a}} r_i \cos\delta + \left(h_j + z\right)^2}} - \frac{1}{\sqrt{R_{\mathrm{a}}^2 + r_i^2 - 2R_{\mathrm{a}} r_i \cos\delta + \left(h_{\mathrm{a}} + z + h_j\right)^2}} \right] d\delta \tag{7}$$

where I is the current, n_{s} and n_{t} are the number of coil turns and layers, respectively, r_i is the radius of ith coil, and h_j is the height gap of the jth coil layer. Magnetic and electromagnetic potential energies were calculated by integrating each force acting on the magnet over the displacement ($\int F\mathrm{d}z$).

We neglect the gravitational force and external damping by ambient air. The gravitational force $F_{\mathrm{g}} = m\,g \approx (10^{-4}\ \mathrm{kg})(10\ \mathrm{m/s^2}) \approx 10^{-3}$ N and the form drag $F_{\mathrm{fl}} = \frac{1}{2} C_D \rho_a A v^2 \approx \frac{1}{2}(1)(1.2\ \mathrm{kg/m^3})(10^{-4}\ \mathrm{m^2})(0.5\ \mathrm{m/s})^2 \approx 10^{-5}$ N are both insignificant compared to other forces, which are on the order of 10^{-1} N. In effect, the structure showed consistent performance in both horizontal and vertical orientations.

Using the defined force components, the equations of motion for the magnet are numerically solved in MATLAB based on Newton's second law:

$$m\ddot{z} = F_{\mathrm{ES}} - F_{\mathrm{MM}} - F_{\mathrm{EM}} - F_{\mathrm{d}}, \tag{8}$$

where m is the mass of a single magnet. The kinetic energy of the magnet was determined using the numerically obtained velocity, given by $\frac{1}{2}mv^2$. The membrane's mass is neglected due to its relatively small contribution. Once the magnets collapse onto the electromagnet surface, we assume an inelastic collision and reset the magnet's velocity to zero. Variations in the coefficient of restitution have minimal influence on the system's overall dynamics, owing to the dominant magnetic attraction. Key experimental parameters, including E, η, M_{a}, and M_{b}, are independently measured and detailed in the Methods section of the main text. The geometric and electrical specifications of the electromagnet used throughout the study are listed in Table 1.

For the system corresponding to Fig. 2, we use elastic membrane of Ecoflex50 of initial thickness 0.33 mm with prestretch $\lambda_{\mathrm{p}} = 1.5$ (final thickness $h_{\mathrm{m}} = 0.147$ mm) and radius $R_{\mathrm{m}} = 10$ mm. The moving magnets have the total thickness $h_{\mathrm{a}} = h_{\mathrm{b}} = 1.5$ mm (by stacking three 0.5 mm magnets) and radii $R_{\mathrm{a}} = R_{\mathrm{b}} = 4$ mm, made of neodymium with

density ρ = 7500 kg/m^3. Extra acrylic spacers of 0.7 mm were attached on both top and bottom of the electromagnet to control peak magnetic forces.

Table 1. Experimental parameters of the electromagnet

Variable name	Value
R_{EM}	0.5 Ω
n_s	11
n_t	4
$r_{i,\mathrm{inner}}$	2.4 mm
$r_{i,\mathrm{outer}}$	6.4 mm
h_1	0.2 mm
h_2	0.6 mm
h_3	1.0 mm
h_4	1.4 mm

Supplementary Note 2. Static force analysis

As shown in Supplementary Fig. 2a, the current applied to the electromagnet produces a corresponding magnetic field. During the half-cycle from the point of maximum repulsive force at t_0 to the point of maximum attractive force at t_2, we analyzed the forces exerted on the magnet. In NC-EsMV, at $t = t_0$, the electromagnet pushes the magnet to its farthest position. At $t = t_2$, the electromagnet pulls the magnet to its closest position (Supplementary Fig. 2b). These forces vary with the distance (z) between the magnet and the electromagnet. Depending on the polarity of the alternating current (AC) input, the electromagnetic force alternates between repulsive ($F_{EM, Rep}$, red) and attractive ($F_{EM,Att}$, green). To intuitively represent the magnet's equilibrium position, we plotted the negative elastic force (-F_{ES}), generated by the stretched membrane, instead of the positive elastic force (F_{ES}) (Supplementary Fig. 2c). The elastic membrane resists the magnet's motion toward the electromagnet and vice versa. The intersections of these force curves, indicated by the black circles at t_0 and t_2, represent equilibrium points where the forces balance. Thus, the magnet in the NC-EsMV system oscillates between these positions. Here, the inertial effects are considered negligible. Consequently, the magnet's equilibrium points shift over time. The measured forces closely match the theoretical predictions (detailed in Supplementary Notes 1), validating our model for the dynamic force interactions in the system.

In C-EsMV system, at $t = t_0$, the magnet reaches its maximum displacement due to repulsion, and at $t = t_2$, it touches the electromagnet's surface because of the attractive forces, as shown in Supplementary Fig. 2d. Although the elastic force (F_{ES}) remains the same, but the magnet-magnet interaction force is added to the previous electromagnetic components ($F_{MM} + F_{EM, Rep}$ and $F_{MM} + F_{EM, Att}$) in Supplementary Fig. 2e. Optimal motion amplification occurs when the negative elastic force curve ($-F_{ES}$) is located between the maximum attraction ($F_{MM} + F_{EM, Att}$) and repulsion ($F_{MM} + F_{EM, Rep}$) curves. When $|-F_{ES}| < |F_{MM} + F_{EM, Att}|$ at t_2, the magnets collapse onto the electromagnet in the middle. As time progresses and $|-F_{ES}| > |F_{MM} + F_{EM, Att}|_{z=0}$ dominates at t_0, the magnet moves back to the balance point. However, due to significant inertia, static analysis alone is insufficient to fully capture the system's behavior. Static analysis inherently assumes equilibrium conditions without considering temporal effects, neglecting events preceding or following the equilibrium state. Thus, a dynamic analysis that explicitly incorporates acceleration and temporal context becomes essential. This dynamic perspective is discussed in detail in Fig. 2.

Supplementary Note 3. Resonant frequency in our system

In our system, the observed resonance corresponds to the nonlinear forced resonance of the coupled magnet–membrane system rather than the intrinsic free-vibration frequency. Consequently, the response around resonance is asymmetric: the vibration amplitude increases gradually as the frequency approaches resonance, but exhibits an eventual decay beyond the resonance frequency. At lower frequencies, the system surpasses the threshold to generate amplified vibration, and as the frequency approaches resonance, this effect further enhances the amplitude. Beyond resonance, however, the increasing phase mismatch between the magnet motion and the input signal reduces effective acceleration, preventing the system from overcoming the threshold and leading to weakened vibration and eventual decay.

This asymmetry primarily arises from the phase mismatch–induced loss of energy transfer efficiency at higher frequencies. In addition, the strongly nonlinear magnetic force, which intensifies as the inter-magnet distance decreases, further enhances this asymmetric response. Together, these effects cause the frequency response to deviate from the symmetric Lorentz-type profile typical of linear oscillators.

The intrinsic baseline frequency of the magnet–membrane pair, in the absence of magnetic and damping forces, can be approximated by the mass–spring relation:

$$\omega_0 = \sqrt{\frac{k_{\mathrm{ES}}}{m}} \tag{9}$$

where k_{ES} is the spring constant of membrane and m is the magnet–membrane mass.

When magnetic and damping forces are included, the system can be linearized locally around an equilibrium position z^*:

$$\begin{gathered} m\,z'' + c_{\mathbf{ef}}\,z' + k_{\mathbf{ef}}\,z \;= 0, \\ k_{\mathbf{ef}} \;= \frac{\delta(F_{\mathrm{ES}} - F_{\mathrm{M\,M}})}{\delta z}_{\text{at z=z}^*}, \end{gathered} \tag{10}$$

The corresponding damped frequency is given by:

$$\omega_{\mathrm{d}} = \sqrt{\frac{k_{\mathbf{ef}}\,(z^*)}{m} - (\frac{c_{\mathbf{ef}}}{2m})^2}, \tag{11}$$

where c_{eff} denotes the effective damping coefficient of the membrane, which in our system may depend not only on velocity but also on displacement due to the viscoelastic membrane behavior and contact conditions. k_{eff} (z^*) varies dynamically with the equilibrium position determined by the applied magnetic forces. Thus, the resonant frequency shifts continuously with both equilibrium position and input current, confirming that the observed resonance arises from a nonlinear forced response rather than a fixed natural frequency.

For small oscillations around an equilibrium z^*, $c(z)$ can be approximated as constant, yielding a well-defined local damped natural frequency. However, in the amplified state, the C-EsMV traverses both equilibria with large excursions, where the restoring and damping forces vary significantly within each cycle. The motion is therefore better described as a nonlinear forced periodic response (limit cycle) governed by the AC electromagnetic drive, whose magnitude depends on displacement while its temporal variation follows the input frequency. This displacement dependence introduces nonlinear stiffness effects, broadening and shifting the resonance relative to eq. (9).

Although the membrane can, in principle, support multiple vibration modes, our experiments operated near the fundamental resonance, where phase mismatch and potential damping effect of membrane strongly suppresses higher-order responses. Weak secondary resonances may appear under certain conditions but are highly sensitive to asymmetry and nonlinear coupling. If geometric or material asymmetry were introduced, multimode or non-axisymmetric oscillations could emerge, leading to richer nonlinear dynamics under modified designs or excitation conditions.

Supplementary Note 4. Design map for optimal region

In extension to Fig. 3a, we further examined the system by analyzing combinations of characteristic magnetic and elastic forces from various perspectives. As shown in Supplementary Fig. 7a, the system enters an *amplified* state (highlighted in yellow) when the membrane force and magnetic force are balanced. However, the range over which amplification occurs (amplifiable range, ΔD) varies under different cases (Supplementary Fig. 7b). The *weakened* state (indicated in blue) dominates when the magnetic force is disproportionately large or small, regardless of the membrane's initial position (D). The maximum amplitude achievable by the system occurs at the optimal initial position (D_{opt}), placed at the boundary between the *amplified* state (or the *collapsed* state, if amplification is not possible) and the *weakened* state.

Supplementary Fig. 7c demonstrates that the D_{opt} increases as the membrane force (F^*_{ES}) decreases and the magnetic force (F^*_{MM}) increases, indicating that stronger magnets can initiate amplification from farther distances by more easily overcoming the membrane's restoring force. Supplementary Fig. 7d and e further show that, at the optimal position (D_{opt}) of each force combination (F^*_{ES}, F^*_{MM}), high velocity and acceleration are observed along an upward-sloping amplification boundary where the elastic and magnetic forces are in balance. Among the systems capable of amplification, those with weaker elastic forces tend to exhibit even higher velocities and accelerations. This is because stronger, less stretchable membranes more severely restrict the magnet's displacement, thereby limiting its dynamic motion.

Moreover, increasing the magnetic force often requires larger magnets, as magnetic materials have finite magnetization. While this increases the magnetic force, it also raises the system's mass, which could reduce acceleration. Nevertheless, as shown in Supplementary Fig. 7f, systems with greater magnetic forces—and consequently larger masses—generally exhibit higher kinetic energies.

Table 2. Characteristic parameters and forces for four cases

Case	Membrane modulus (kPa)	Magnet thickness (mm)	F^{*}_{ES} (N)	F^{*}_{MM} (N)
I	50	0.5	0.2419	0.0348
II	50	1.5	0.2419	0.2072
III	110	1.5	0.6289	0.2072
IV*	80	2.2	0.3870	0.3463

All other parameters remain unchanged. *Simulated

Supplementary Note 5. Characterization of C-EsMV based on the initial magnet position, *D*.

The C-EsMV can exhibit three distinct states—*collapsed*, *amplified*, and *weakened*—based on the relative positions of the magnets, electromagnet, and membrane. We numerically analyzed these states by fixing all parameters except the initial magnet position (*D*) relative to the electromagnet surface (Supplementary Fig. 9a). Adjusting *D* shifts the membrane's equilibrium position, shown in parallel displacements of the membrane force F_{ES} along the x-axis (from ① to ④ in Supplementary Fig. 9b).

In the *collapsed* state (region including ①), the membrane is so close to the electromagnet that the combined force of magnet attraction and maximum electromagnet repulsion (F_{MM} + $F_{EM, Rep,}$ red line) exceeds the elastic force of membrane at the bottom surface. When $|F_{ES}(z = 0)| < |F_{MM}(z = 0) + F_{EM, Rep}(z = 0)|$, the magnet remains attached and cannot detach, indicating a *collapsed* configuration.

In the *amplified* state (region including ②), the membrane force curve $-F_{ES}$ lies between the repulsive and attractive force curves (red and green lines). In this regime, $-F_{ES} > F_{MM} + F_{EM, Att}$ for all *z,* and $|F_{ES}(z = 0)| > |F_{MM}(z = 0) + F_{EM, Rep}(z = 0)|$, leading to a greatly amplified stroke between the bottom surface and the upper equilibrium position.

In the *weakened* state (regions including ③ and ④), the membrane force curve, $-F_{ES}$, is below $F_{MM} + F_{EM, Att}$ for some point ($\exists\, z$ such that $-F_{ES} < F_{MM} + F_{EM, Att}$), meaning the magnet is not sufficiently displaced toward the bottom, and the system fails to reach the amplified stroke.

The *weakened* state can be further subdivided into *shootable* (area including ③) and *non-shootable* (area including ④) regimes, based on whether the actuator can be externally triggered into the amplified state. In the *shootable* regime, the condition $-F_{ES} > F_{MM} + F_{EM, Att}$ creates an energy barrier. However, amplification can still occur by manually bringing the magnets into contact with the electromagnet at least once. The initial attachment allows the system to sustain amplification via magnet's inertia, which helps overcome the energy barrier in subsequent cycles.

In contrast, the *non-shootable weakened* state occurs when the membrane is too far from the electromagnet. Here, the magnetic attraction and maximum repulsion are both weaker than the membrane force at $z = 0$ ($|F_{ES}| < |F_{MM}$ +

$F_{EM, Rep}$|), preventing any contact between the magnet and electromagnet. As a result, the system experiences damped vibrations and cannot initiate amplification. This highlights the importance of dynamic analysis, as static force conditions alone do not capture the possibility of induced motion.

The total force and energy profiles in Supplementary Fig. 9c-f illustrate the key characteristics of each state at representative D values (2.5, 3.9, 4.5, 8.0 mm) during both attractive and repulsive phases. In the *collapsed* state, the energy barrier persists throughout both phases, preventing detachment from $z = 0$ (Supplementary Fig. 9c). In the *amplified* state, this barrier vanishes, and energy landscapes near the bottom are monotonic in both phases, enabling alternating attachment and detachment (Supplementary Fig. 9d). In the *shootable weakened* state, the energy barrier during the attractive phase indicates the minimum trigger energy required for amplification (Supplementary Fig. 9e). The corresponding force profile shows the minimum trigger force and the maximum bearable load, defined as the heaviest object the C-EsMV system can support without losing its amplification capability. Lastly, in the *non-shootable weakened* state, the energy barrier is absent in both phases, producing a profile similar to that of NC-EsMV (Supplementary Fig. 9f).

Supplementary Note 6. Efficiency of the system

To evaluate the performance of the C-EsMV system, we compare the efficiency under various input waveforms relative to that under a sinusoidal input. The absolute efficiency is defined as the ratio of output kinetic energy to input electrical energy and it is relatively low (~1%) when driven by sinusoidal currents through electromagnets. However, this value is not fixed; rather, it can be substantially enhanced from two perspectives.

First, the magnetic field generated by the electromagnet is given by $B = \mu n i$, where n is the number of coil turns and i is the current. The current low efficiency of the C-EsMV system is primarily attributed to the use of a small and lightweight electromagnet (1 g, 40 turns), which necessitates a high driving current. Efficiency can be improved by increasing the number of the coil turns (n), thereby reducing the required current and, thus, the input electrical energy. Since this system is intended for stationary applications, where weight is not a limiting factor, this trade-off would be acceptable for practical scenarios. Crucially, high current itself does not benefit the system; rather, amplification is triggered once a threshold current is surpassed. Lowering this threshold allows the system to operate at lower input currents, thereby enhancing overall efficiency and broadening its application potential.

The second approach involves maintaining the same peak current required for amplification while modifying the input wave form, as shown in Fig. 3i. Since electrical energy input corresponds to the time-integral for voltage (or current), minimizing the area under the input signal while still reaching the amplification threshold significantly improves efficiency. We demonstrate that using a pseudo-Gaussian waveform, with the same electromagnet and peak current, achieves amplification with a 64-fold increase in efficiency compared to a sinusoidal input. Further optimization of the waveform to achieve smaller integral area with a sharp, brief peak could improve efficiency even more.

Optimizing either the electromagnet structure or the input waveform presents a promising route for enhancing the absolute efficiency of the C-EsMV system. Nonetheless, the primary contribution of this study is to demonstrate that meaningful motion amplification can be achieved by balancing magnet-magnet interaction through elastic energy. We further envision that the electromagnet could be replaced with alternative actuation methods, such as electrical or pneumatic actuators, that can similarly balance two key forces (F_{MM}, and F_{ES}) for improved adaptability and energy performance. While these engineering refinements are important, the present work focuses on establishing the fundamental principle underlying elastic-mediated magnetic amplification.

Supplementary Figure:

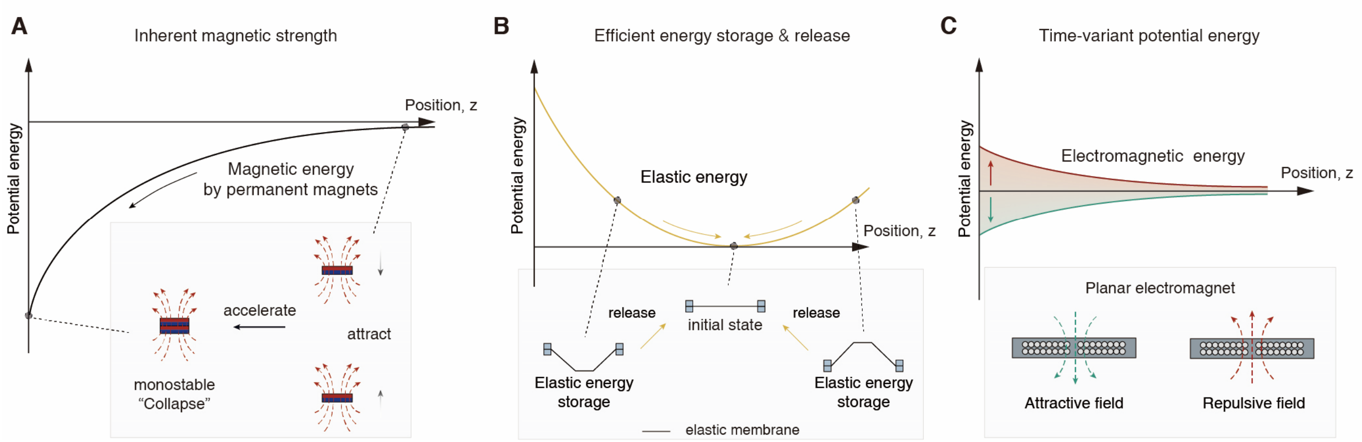

Supplementary Fig. 1. Schematic representation of potential energy in (a) attractively coupled magnets, (b) an elastic membrane, and (c) an electromagnet.

In (a), two like-pole magnets accelerate toward each other until they collapse into a stable configuration (black line), reflecting their inherent magnetic strength. In (b), the introduction of an elastic membrane allows the magnetic collapse to be controlled, with the membrane efficiently storing and releasing energy as elastic potential energy. In (c), a planar electromagnet generating an alternating magnetic field enables the creation of a dynamic system with tunable instability.

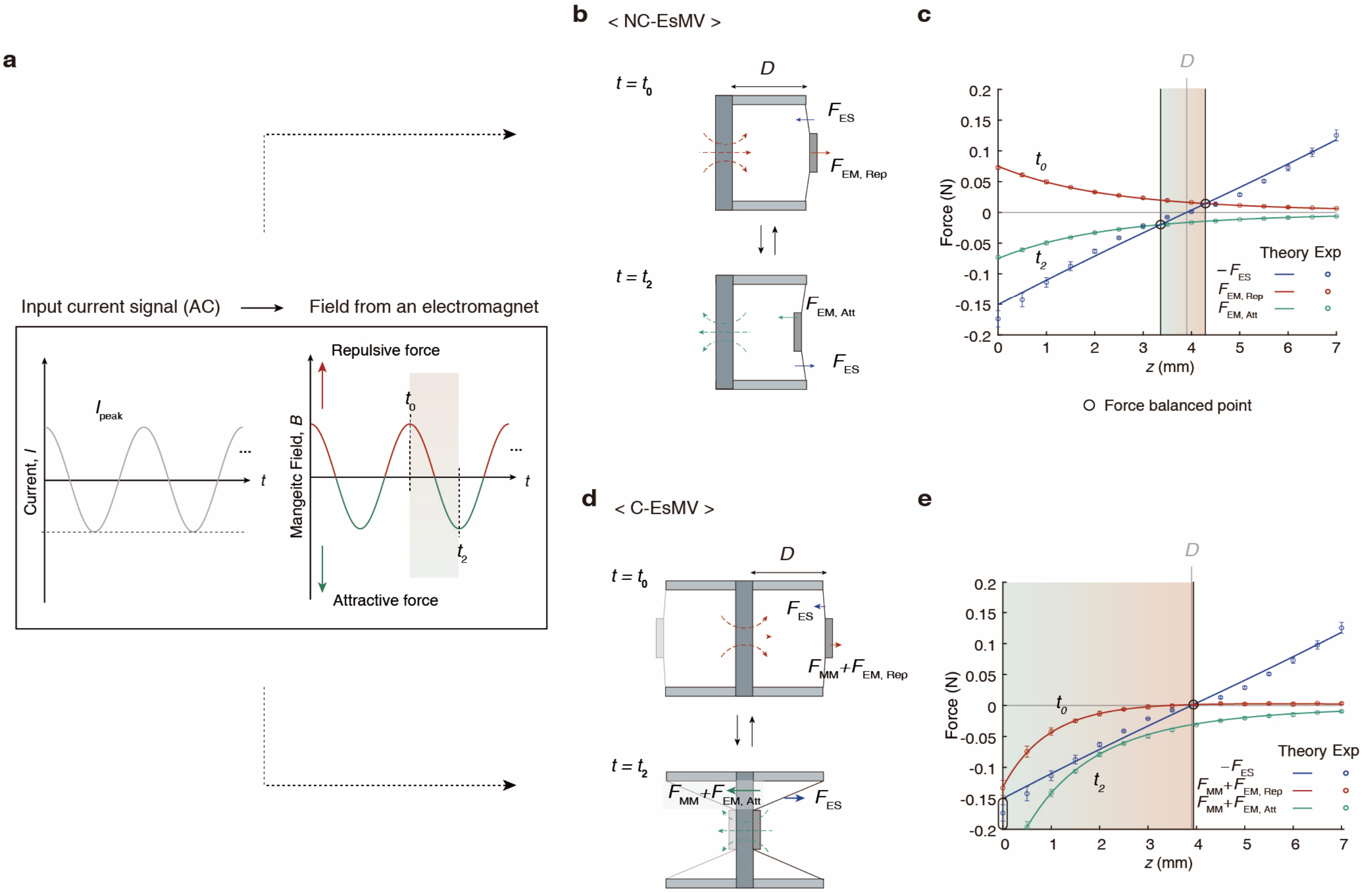

Supplementary Fig. 2. Static force analysis of non-coupled (NC-EsMV) and coupled (C-EsMV) systems.

(a) Input current applied to the electromagnet and the resulting magnetic field generated. (b) The position of the magnet during vibration at $t = t_0$ and $t = t_2$ in NC-EsMV system. (c) A static force diagram showing the individual forces acting on the magnet, including the elastic force (F_{ES}) and electromagnetic forces ($F_{EM,\ Att}$ and $F_{EM,\ Rep}$). In the NC-EsMV system, the magnet oscillates between two force balance points, with the inertial effect considered negligible. (d) The position of the magnet during vibration at $t = t_0$ and $t = t_2$ in C-EsMV. (e) Static force diagram for C-EsMV system, where the magnetic forces are plotted as the sum of the electromagnetic components ($F_{MM} + F_{EM,\ Rep}$ and $F_{MM} + F_{EM,\ Att}$). While the static analysis shows the magnet oscillating between two balance points, the significant inertia in the system requires a dynamic analysis for a more accurate interpretation, which is detailed in Fig. 2. Error bars denote SDs; n = 3.

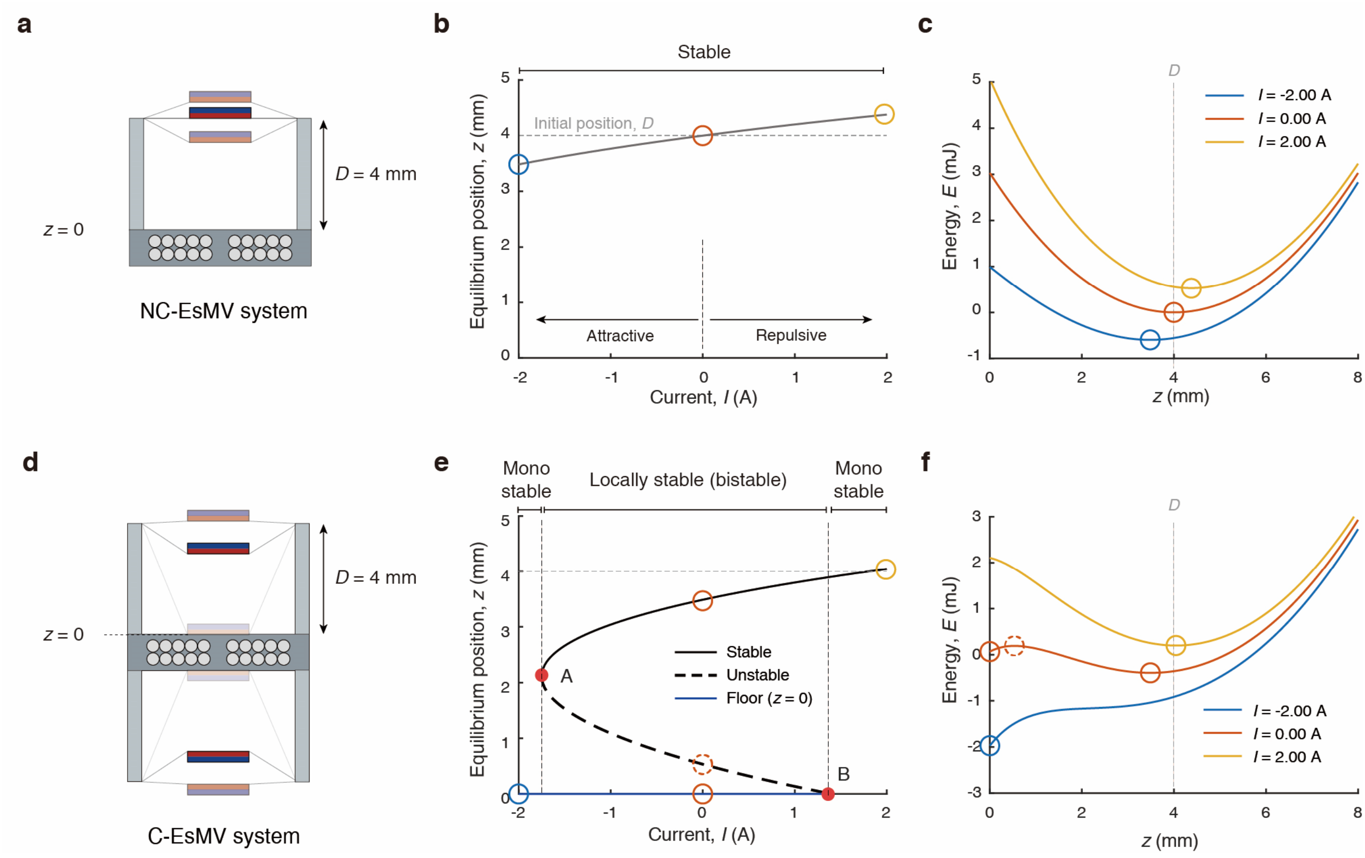

Supplementary Fig. 3. Static analysis of equilibrium and energy as current is varied.

(a) Schematic of the NC-EsMV with variable current (*I*). (b) State diagram showing equilibrium positions of the NC-EsMV system as a function of input current. (c) Energy landscape of the NC-EsMV system plotted as a function of position for different input currents (−2, 0, and 2 A). (d) Schematic of the C-EsMV. (e) State diagram showing equilibrium positions of the C-EsMV system as a function of input current. (f) Energy landscape of the C-EsMV system plotted as a function of position for different input currents (−2, 0, and 2 A) (Point A: Saddle-node fold, Point B: Subcritical Hopf).

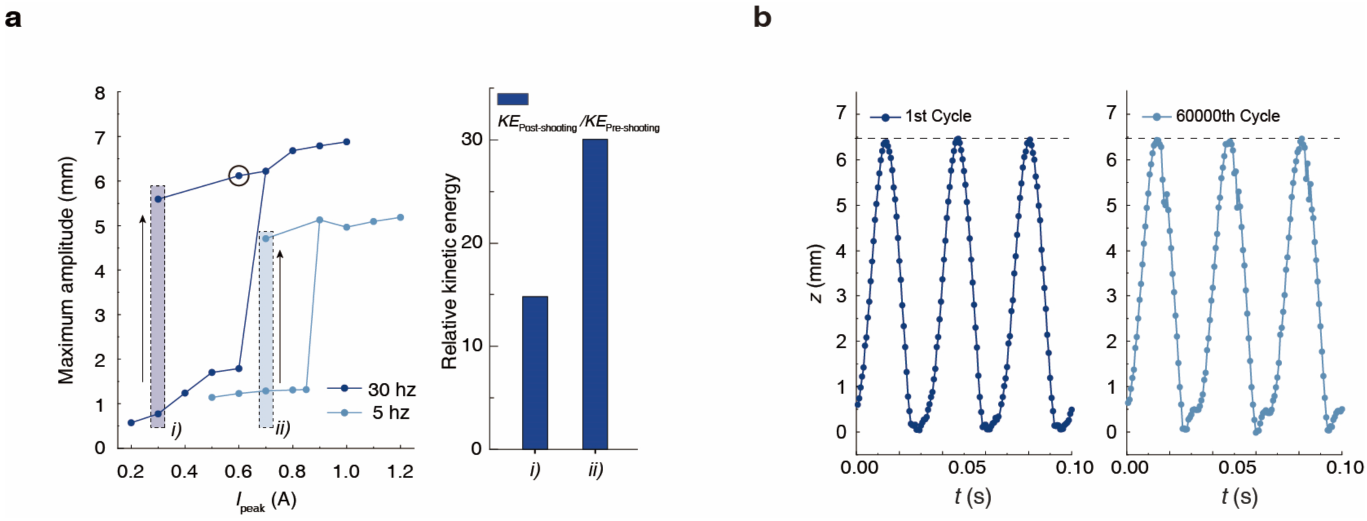

Supplementary Fig. 4. Energy conversion efficiency and cyclic stability of vibrational hysteresis.

From the vibrational hysteresis shown in Fig. 1g, two key properties are measured. (a) The ratio of kinetic energy of the shooted mode to that of non-shooted mode was evaluated for each condition in C-EsMV system: (i) 0.3 A at 30 Hz and (ii) 0.7 A at 5 Hz. The energy gain with and without shooting was assessed, showing a significant increase when shooting is utilized. (b) Cyclic stability of shooting at 0.6 A and 30 Hz. Even after 60,000 cycles, stable behavior was observed, demonstrating the system's robust cyclic stability.

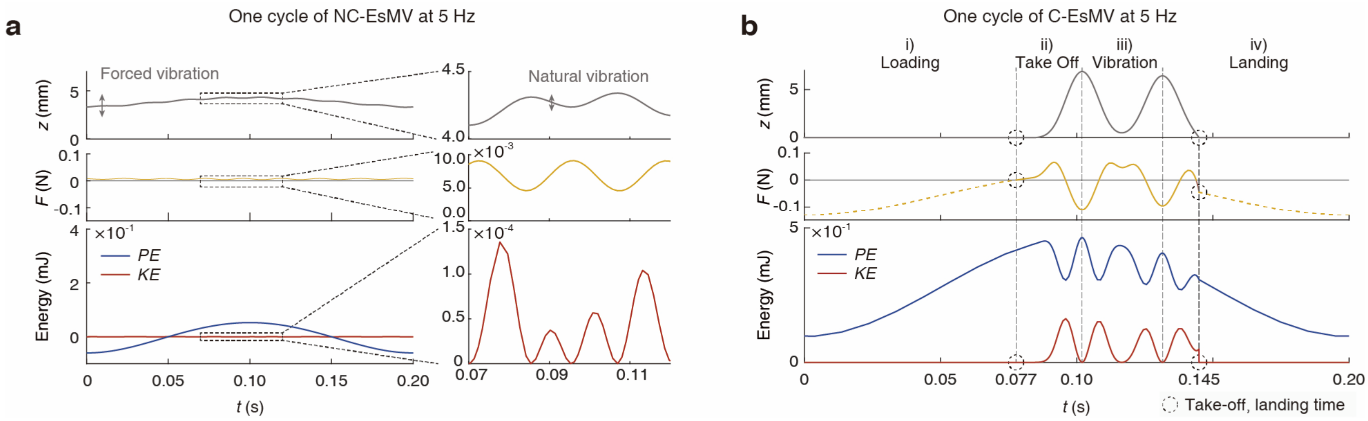

Supplementary Fig. 5. Numerical results for magnet's position (*z*), net force (*F*), and potential and kinetic energies during a single actuation cycle of (a) NC-EsMV and (b) C-EsMV at f_i = 5 Hz.

Key moments, such as *take-off* and *landing*, are highlighted with dashed circles, and dashed curves are used in the force plot to indicate the zero-net-force regimes. *i) loading, ii) take-off, iii) vibration,* and *iv) landing*, marked by vertical dashed lines. Initially, the magnet collapses onto the electromagnet's surface, storing maximum elastic potential energy (*i*, $0 \text{ s} < t < 0.077 \text{ s}$). When electromagnetic repulsion increases sufficiently to yield a net positive force, it triggers the *take-off* (*ii*, $t = 0.077 \text{ s}$). After *take-off*, the system enters a vibration phase governed by its natural frequency (*iii*, $0.102 \text{ s} < t < 0.132 \text{ s}$), then returns to the electromagnet's surface to complete the cycle (*iv*, $0.132 \text{ s} \leq t \leq 0.2 \text{ s}$).

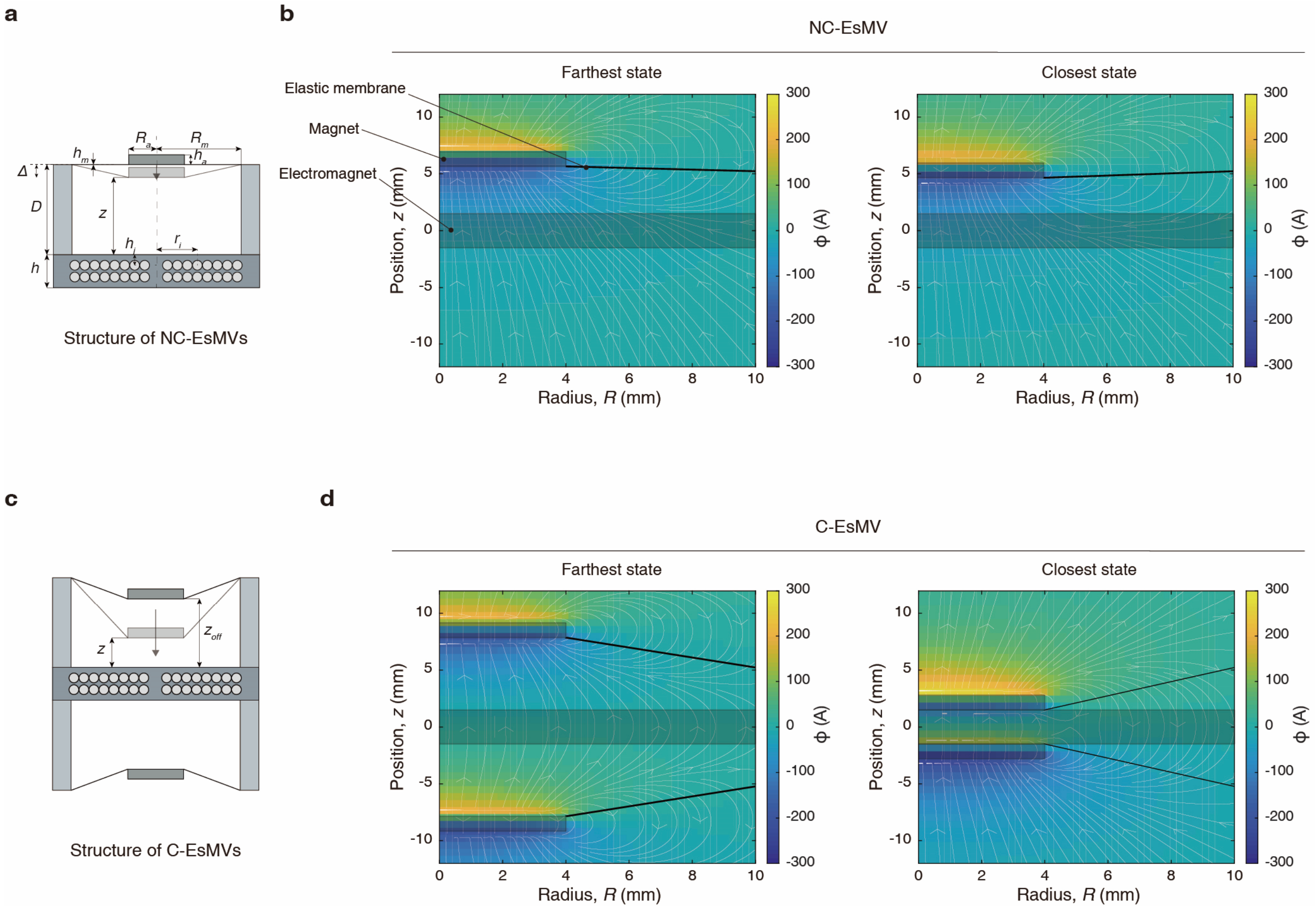

Supplementary Fig. 6. Structural parameters and magnetic scalar potential in the farthest and closest positions of the magnet from the electromagnet phases, for (a and b) NC-EsMV and (c and d) C-EsMV systems.

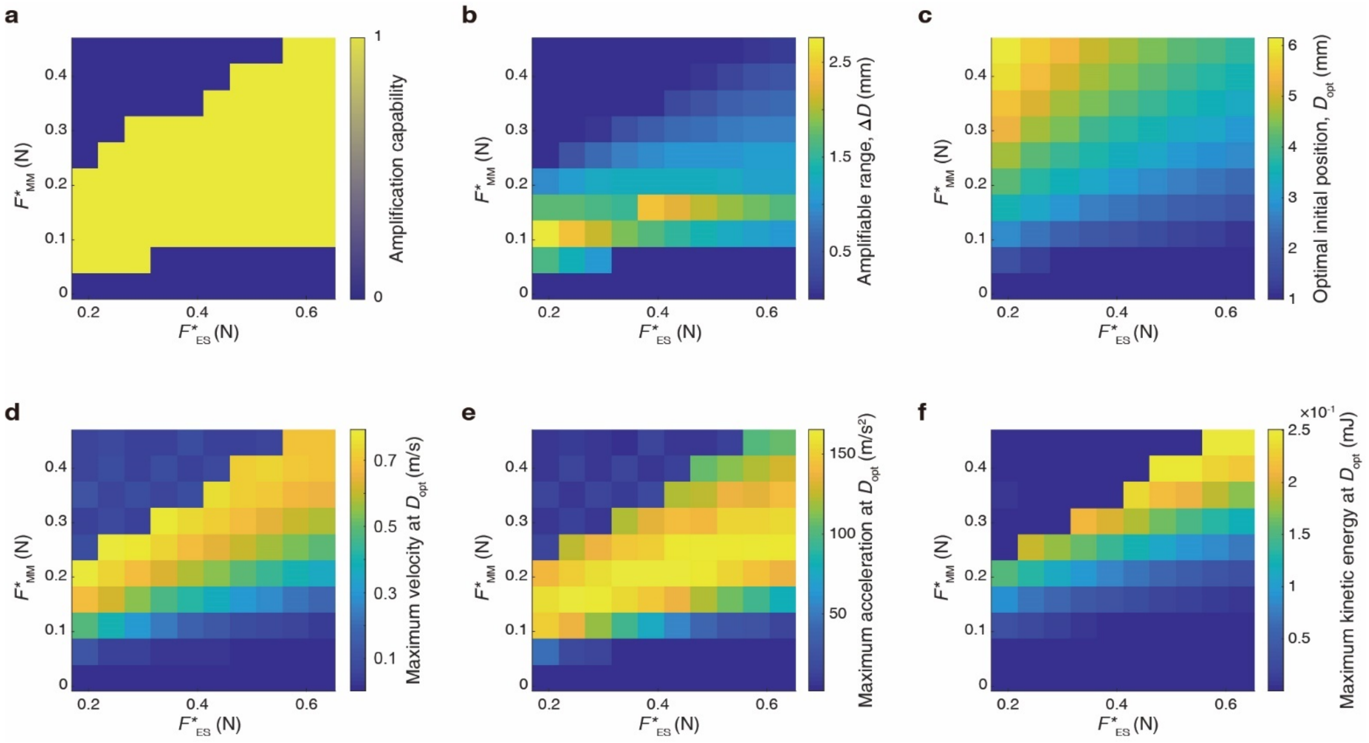

Supplementary Fig. 7. Design map for different cases with varying elastic and magnetic forces. (I_{peak} = 2.0 A)

(a) Amplification capability (yellow: amplifiable, blue: non-amplifiable), (b) range of amplifiable initial positions (D), (c) optimal initial position (D_{opt}), (d) maximum velocity, (e) maximum acceleration, (f) maximum kinetic energy of C-EsMV plotted against characteristic values F^*_{ES} and F^*_{MM}.

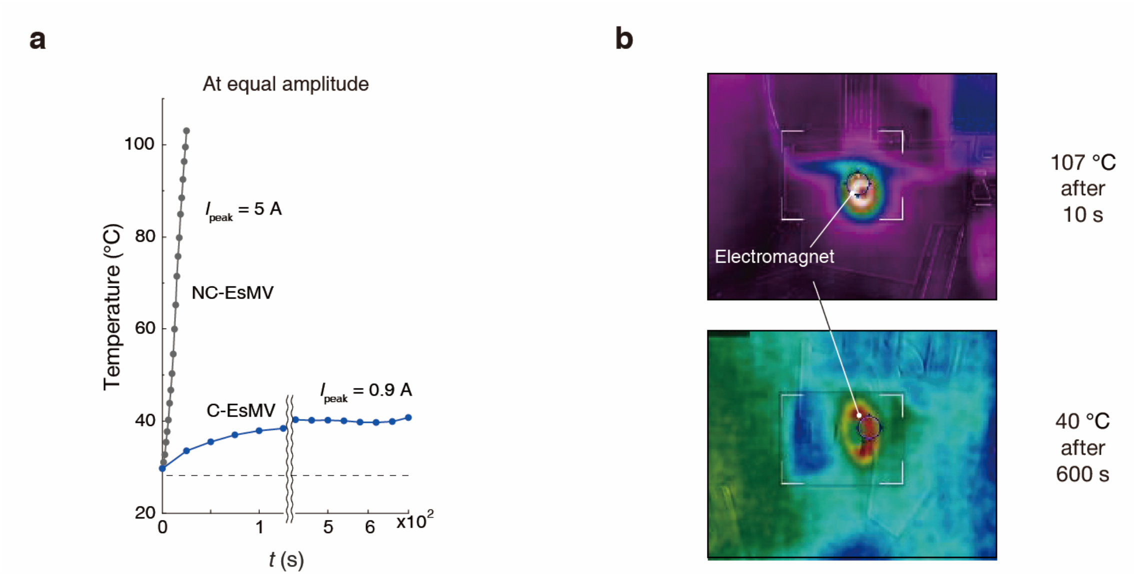

Supplementary Fig. 8. Comparison of thermal stability for each system.

A thermocouple attached to the surface of the electromagnet was used to monitor the real-time temperature during the operation of both NC-EsMV and C-EsMV. (a) For equal amplitude vibrations (D = 2.5 mm), the temperature in NC-EsMV rises exponentially from room temperature to 107 °C within 10 s, while in C-EsMV, the temperature stabilizes at 40 °C, even after 600 s of vibration, demonstrating superior thermal stability due to higher energy efficiency compared to NC-EsMV. (b) Infrared camera images showing the temperature distribution for each case.

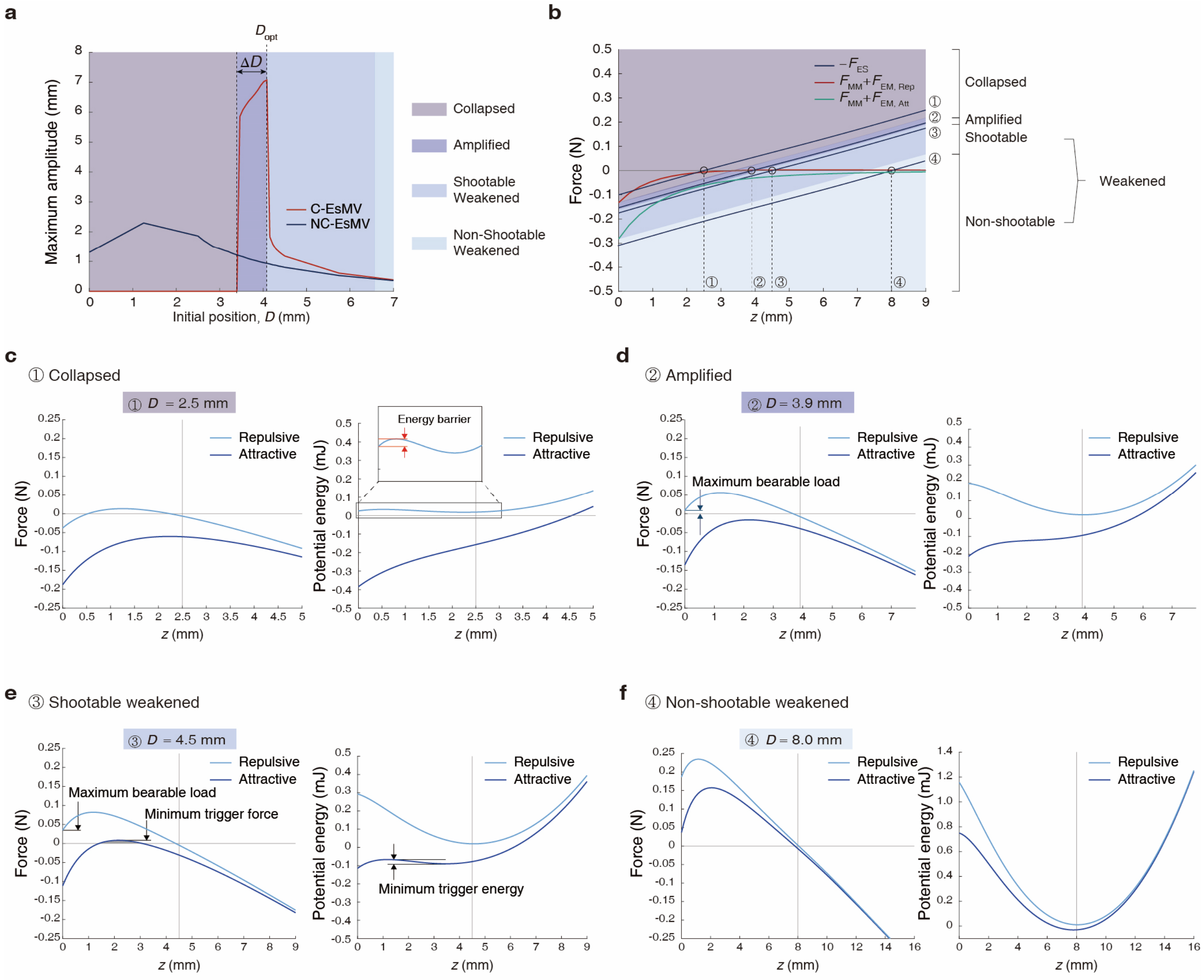

Supplementary Fig. 9. Characterization of C-EsMV based on the initial position *D*.

(a) Initial position-amplitude plot and (b) displacement-force plot of a C-EsMV across varying initial positions, divided into four different regimes. The total force and energy by position for each regime, (c) collapsed, (d) amplified, (e) shootable weakened, and (f) non-shootable weakened highlights the behavior of C-EsMV under various scenarios.

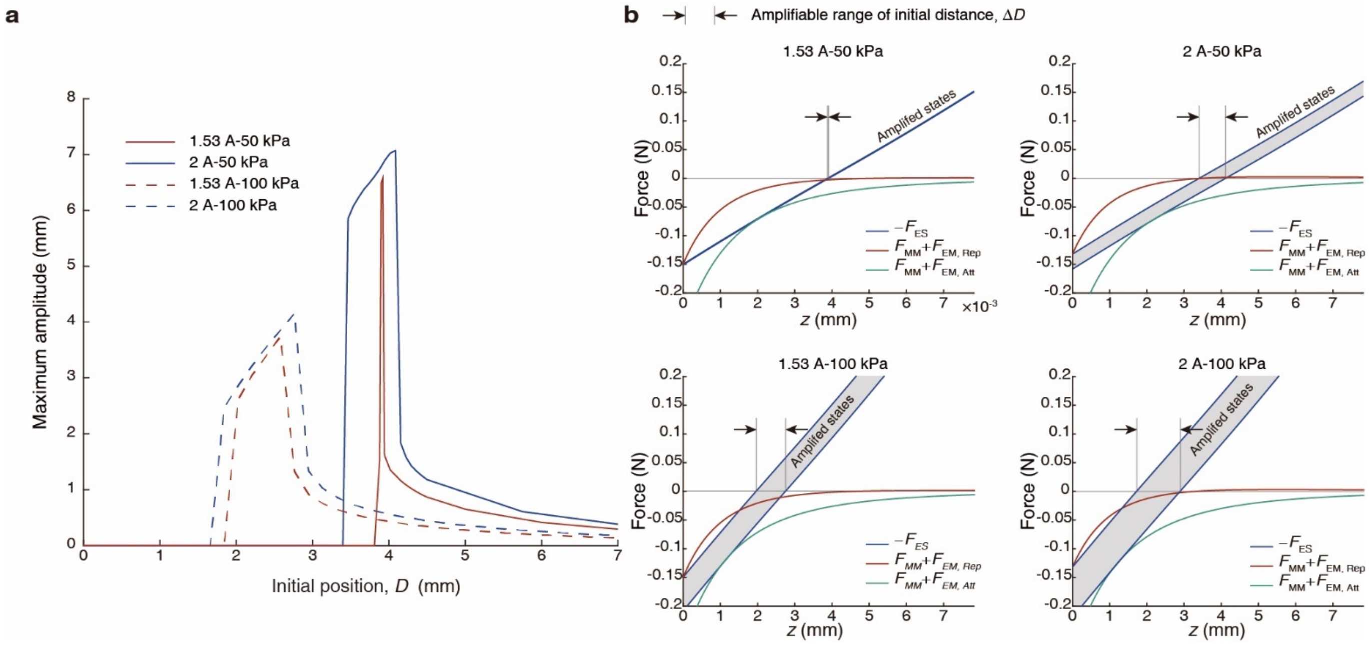

Supplementary Fig. 10. Comparison of four different C-EsMV systems with varied current (I_{peak} = 1.53, 2 A) and membrane modulus (E = 50, 100 kPa) conditions.

(a) Initial position-amplitude and (b) displacement-force plot of the four systems. The shaded area indicates the range of positions where amplification is possible. A higher electric current and a higher elastic modulus expand the amplification range, while a stronger membrane force results in the magnet being positioned closer to the electromagnet.

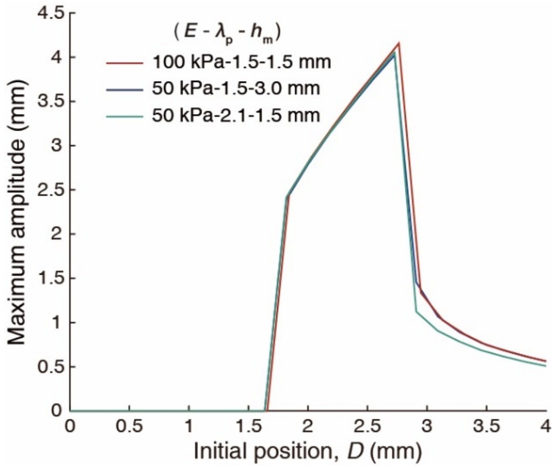

Supplementary Fig. 11. Initial position-amplitude plot of C-EsMV system with varying conditions of elastic membrane, but with identical characteristic membrane force F^*_{ES}.

The three elastic membranes are each specified by different combinations of Young's Modulus E (kPa), prestretch λ_p, and final membrane thickness h_m (mm): (100, 1.5, 1.5), (50, 1.5, 3.0), and (50, 2.1, 1.5). These configurations ensure a consistent characteristic force, F^*_{ES} = 0.49 (N). When F^*_{ES} remains unchanged, the C-EsMV demonstrate similar performance outcomes, highlighting the system's scalability despite variations in membrane physical properties. Any slight deviations observed are due to the nonlinear higher-order effects of the membrane force and the damping force.

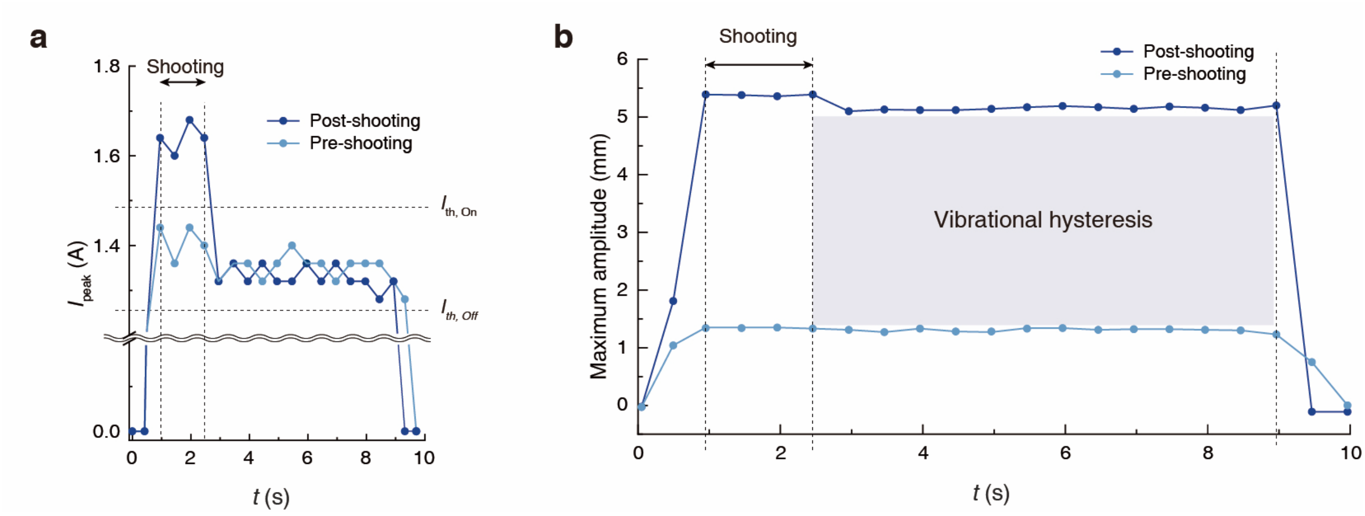

Supplementary Fig. 12. Experimental verification of vibrational hysteresis in C-EsMV.

(a) Input current signal for the two cases: pre-shooting (I_{peak} ~ 1.4 A) and post-shooting (I_{peak} ~ 1.4 → 1.6 A → 1.4 A). (b) Maximum amplitude for each case. When using shooting mechanism, even when the signal returns to the original peak current, the amplification is maintained. The shaded area represents energy saved through vibrational hysteresis, facilitated by inertia during shooting.

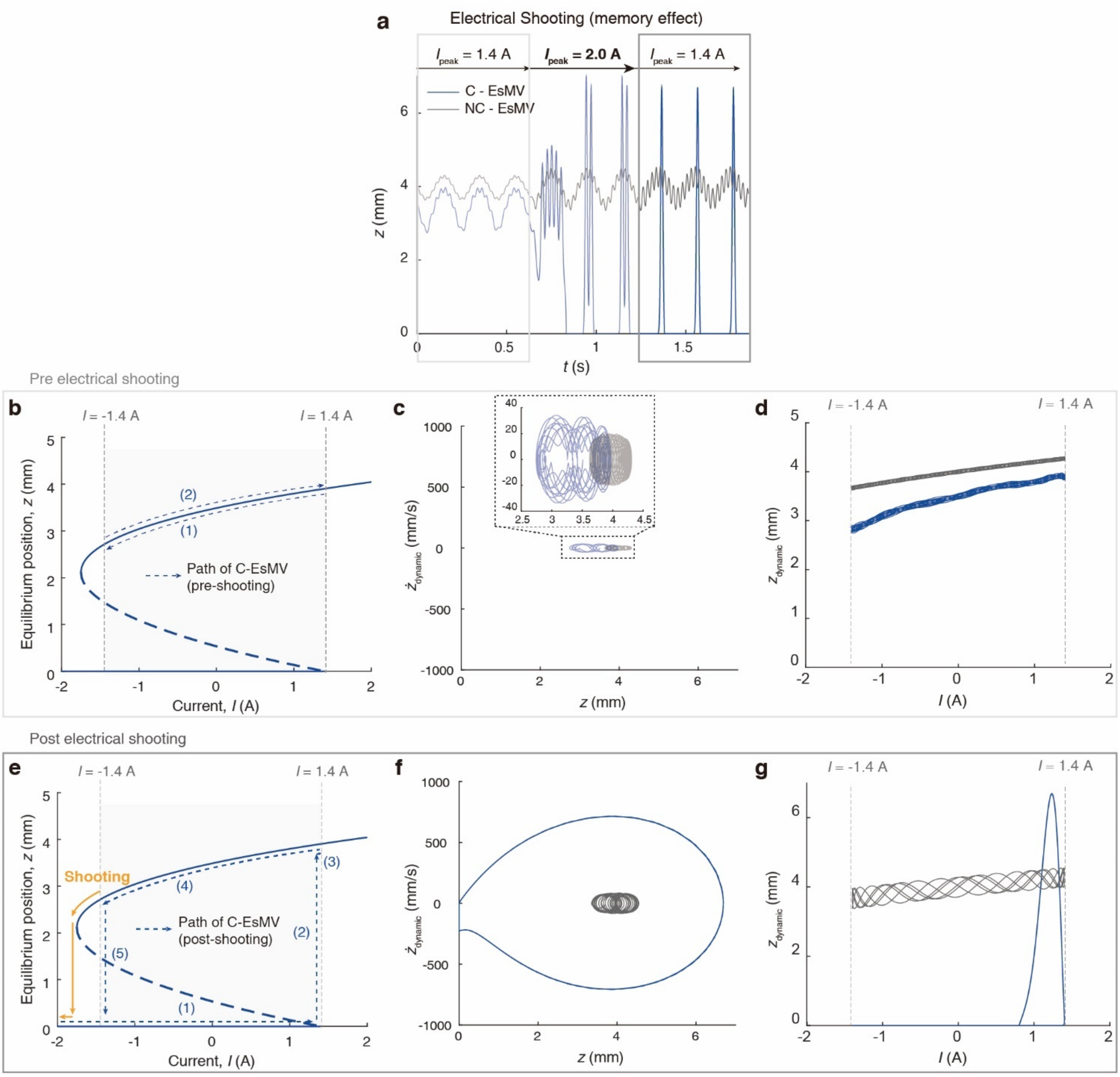

Supplementary Fig. 13. Numerical results of electrical shooting behavior and motion hysteresis in C-EsMV.

(a) Vibration motion of NC-EsMV and C-EsMV at a driving frequency of 5 Hz (D = 4.0 mm). Even after the current is increased from 1.4 A to 2.0 A and then returned to 1.4 A, the amplified vibration of C-EsMV is maintained. The shooting process enables C-EsMV to reach the amplified mode at lower currents that were previously unattainable, as clearly seen in the phase portraits. (b–d) Static and dynamic vibration paths at I_{peak} = 1.4 A before shooting: (b) Bifurcation diagram of C-EsMV showing equilibrium positions, (c) corresponding phase portraits of C-EsMV and NC-EsMV, and (d) dynamic vibration trajectory under sinusoidal electrical input, which closely follows the equilibrium path. (e–g) Static and dynamic vibration paths at I_{peak} = 1.4 A after shooting: (e) Bifurcation diagram of C-EsMV showing a switched branch after shooting, (f) phase portraits of C-EsMV and NC-EsMV, and (g) dynamic vibration trajectory under repeated sinusoidal input, exhibiting significantly larger oscillations despite the same current amplitude.

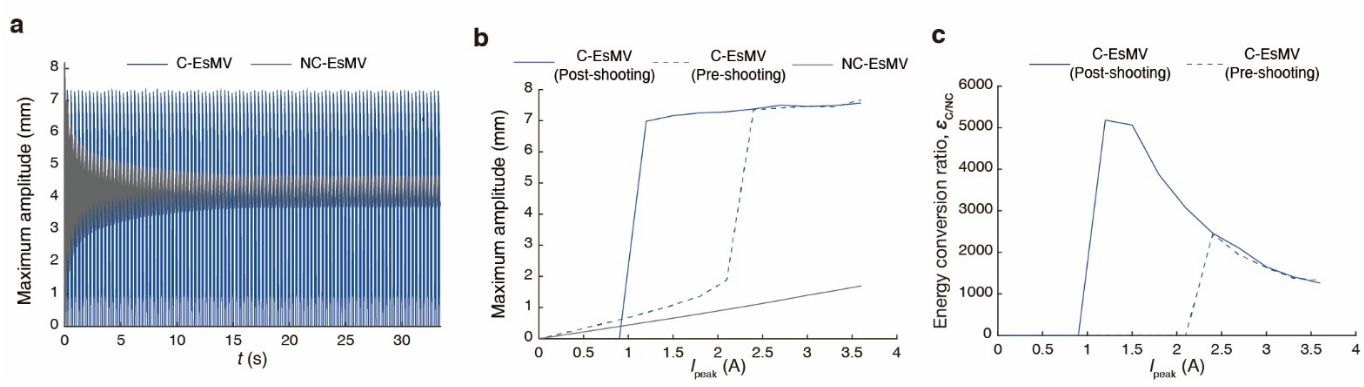

Supplementary Fig. 14. Stability and efficient energy conversion after shooting.

(a) Temporal evolution of NC-EsMV and C-EsMV after the initial shooting (D = 4.2 mm). The vibration in NC-EsMV system quickly decays due to internal damping, while amplification in C-EsMV persists over time. (e) Maximum amplitude and (f) coupling-to-non-coupling energy conversion ratio ($\varepsilon_{\mathrm{C/NC}}$) as a function of input current for NC-EsMV and C-EsMV before and after shooting. The results demonstrate that C-EsMV achieves higher efficiency than NC-EsMV under the same low-current conditions, and that post-shooting operation further enhances efficiency compared to the pre-shooting state within the same C-EsMV system.

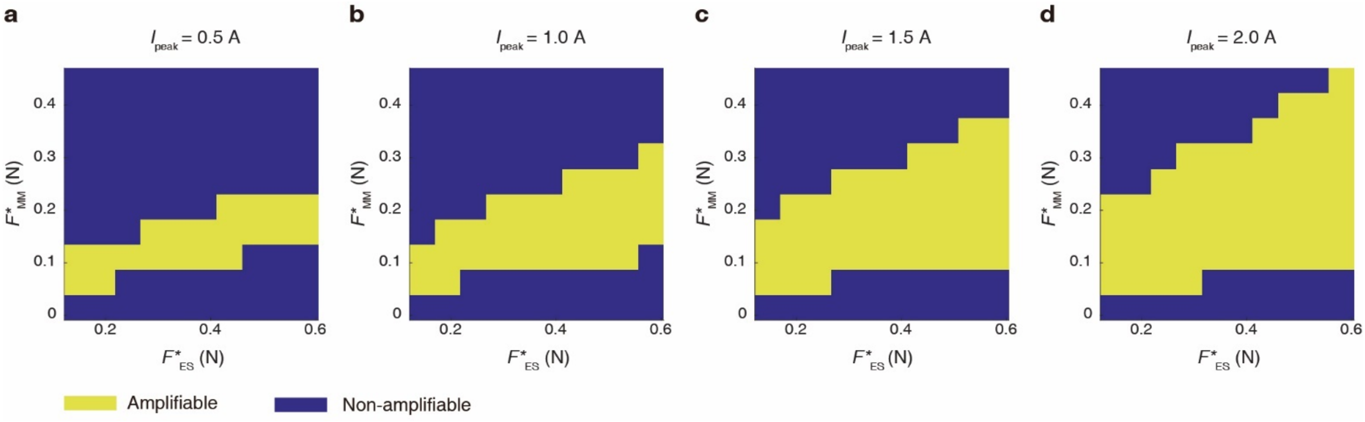

Supplementary Fig. 15. Change in amplifiable regions based on electromagnet input current at I_{peak} of (a) 0.5 A, (b) 1.0 A, (c) 1.5 A, and (d) 2.0 A.

As the current increases, both repulsive and attractive forces become stronger, enabling amplified vibrations over a wider range of magnetic-elastic force combinations.

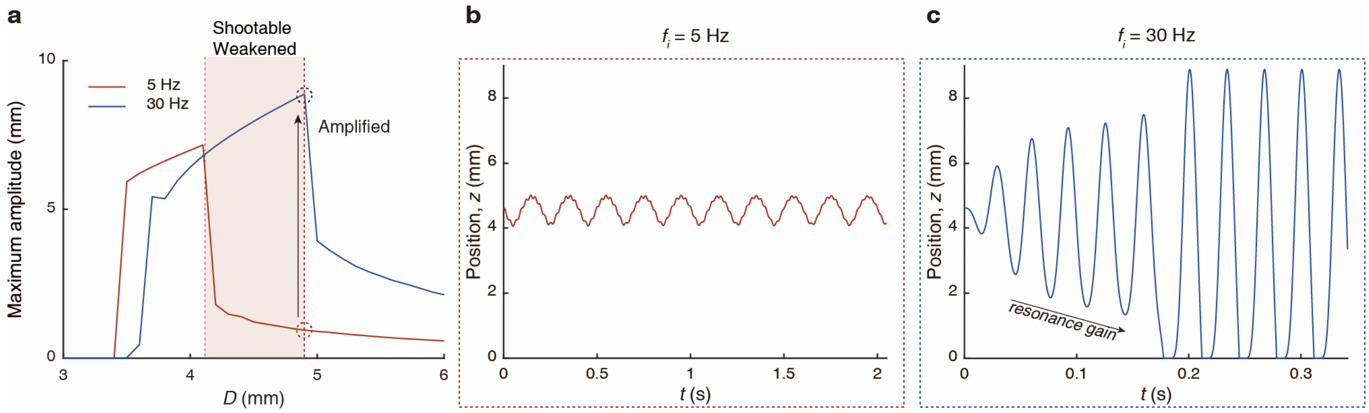

Supplementary Fig. 16. Vibration behavior in a C-EsMV system at different frequencies.

(a) Modulating input frequencies (f_i = 5 Hz to 30 Hz) extends the amplifiable range from the 'shootable weakened' regime to the 'amplified' regime. (b and c) Vibration of each C-EsMV system measured at the optimal distance, D_{opt}, at 5 and 30 Hz.

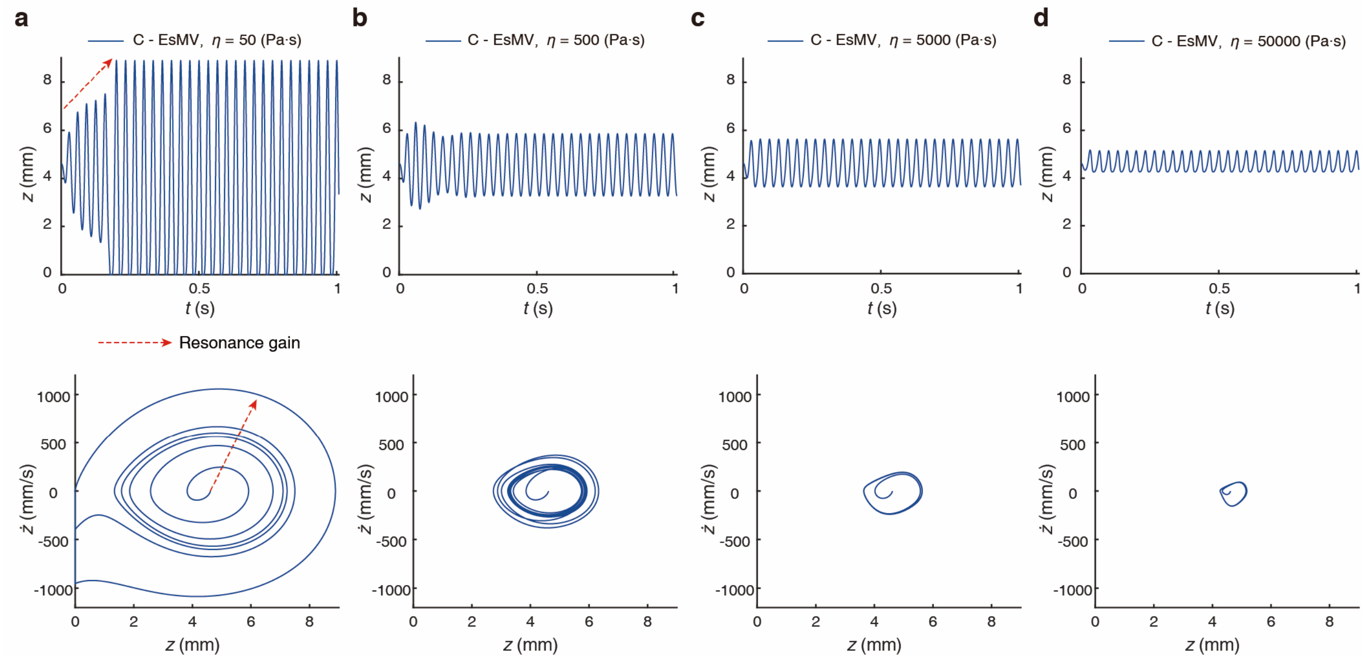

Supplementary Fig. 17. Time-resolved dynamic analysis of membrane motion under resonance at different dynamic viscosities.

(a-d) Dynamic responses when the input frequency is shifted from 5 Hz to 30 Hz, showing the effect of resonance gain at various viscosities: (a) η = 50 Pa·s, (b) η = 500 Pa·s, (c) η = 5000 Pa·s, and (d) η = 50,000 Pa·s.

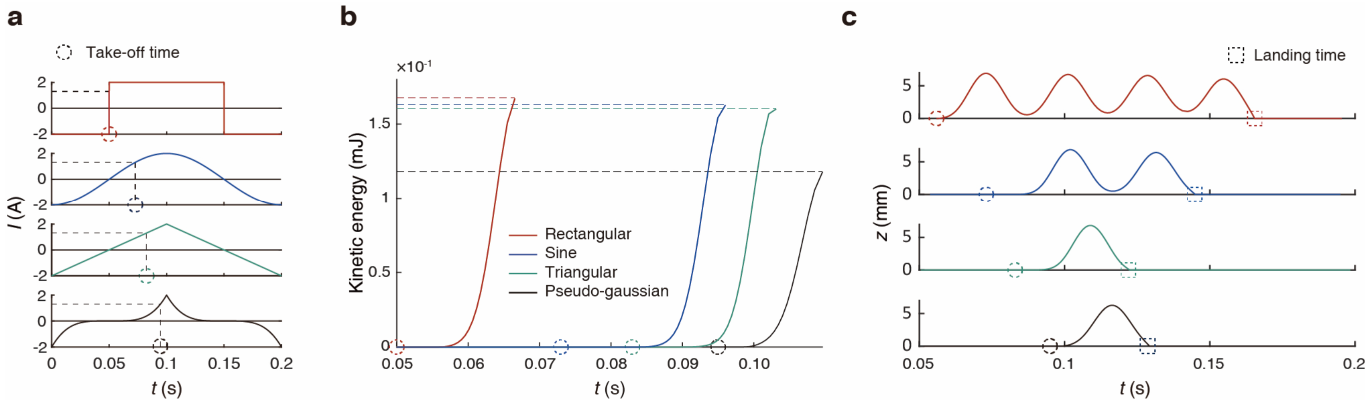

Supplementary Fig. 18. Vibration behavior in a C-EsMV system with different waveforms.

(a) Four types of waveforms: rectangular, sinusoidal, triangular, and pseudo-Gaussian are illustrated. Dotted circle marks the take-off moment of the magnet. (b) Kinetic energy over time during take-off. The rectangular waveform reaches the threshold current quickly, resulting in faster take-off. Differences in kinetic energy arise from varying input energies across waveforms. (c) Full cycle duration (from loading to landing) for each waveform. Both take-off and landing times differ depending on the waveform used.

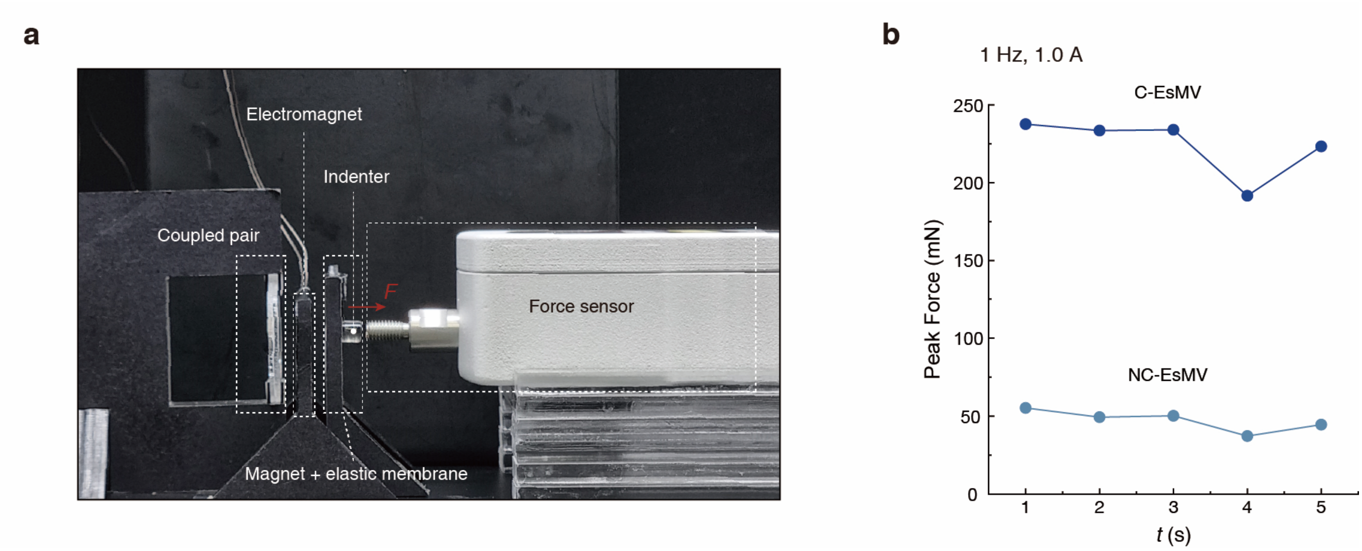

Supplementary Fig. 19. (a) Experimental setup for impact force measurement and (b) real-time peak force at 1 Hz.

A force meter (MARK-10, series 5) was used to measure the impact force during actuation. The system reaches its maximum velocity near the initial membrane position (D), where the membrane stretch is minimal. Therefore, the force sensor was positioned at D for the accurate measurement. Similarly, a thin glass wall (0.1 mm) was set at this position. The average value of five peak forces over time was used to represent the peak impact force.

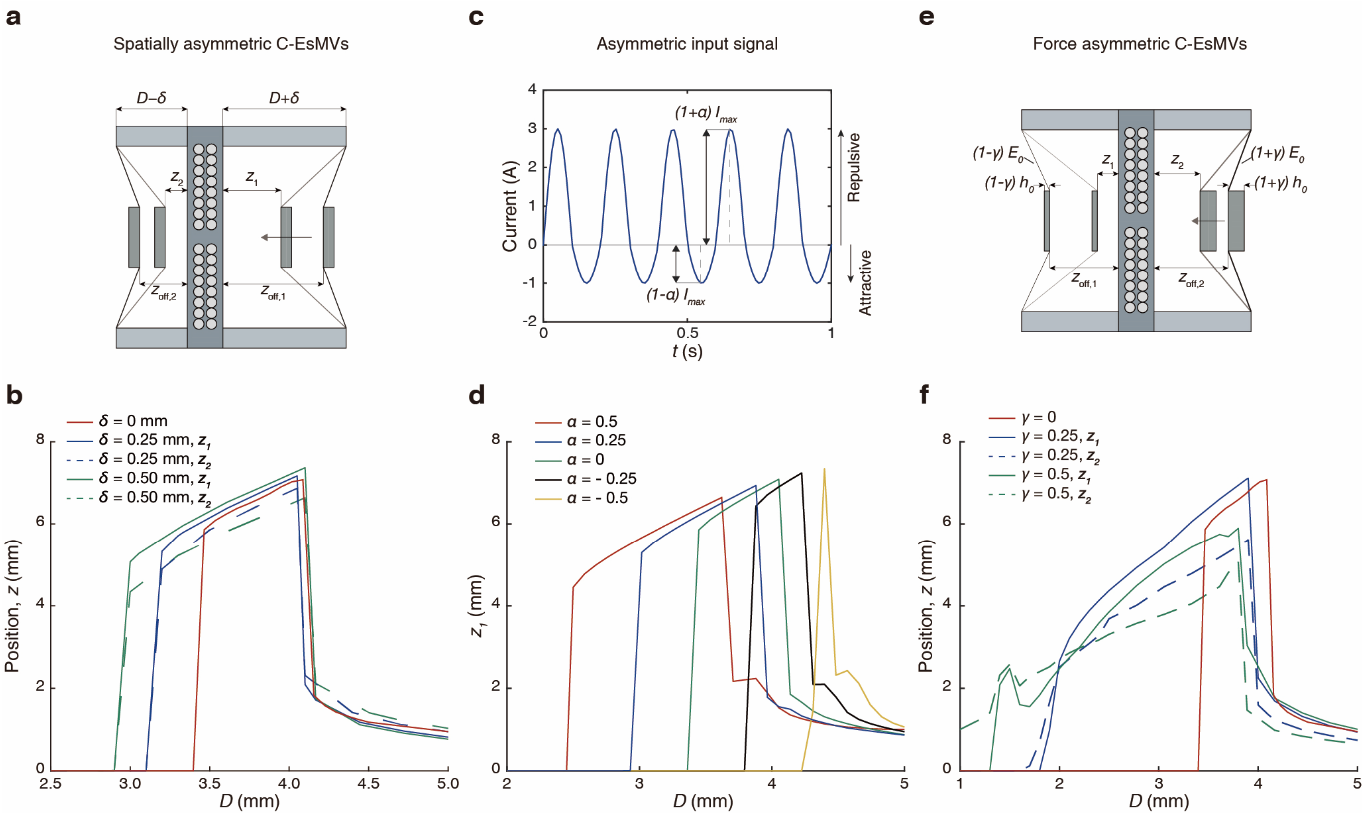

Supplementary Fig. 20. The effect of anisotropy on amplified vibration.

(a) Asymmetric structure and (b) its amplified motion as a function of distance when structural anisotropy is controlled by δ. As δ increases, one magnet moves closer to the electromagnet, making amplification easier and expanding the amplifiable region. The thicker spacer side (z_1) shows a larger amplitude.

(c) Asymmetric waveform input current and (d) its amplified motion as a function of distance when waveform anisotropy is controlled by α, which is defined as the percentage difference between the positive and negative peaks of the AC input: positive peak = (1+ α), negative peak = (1- α). A lower α (stronger attraction) allows amplification at greater distances, but narrows the amplifiable region after collapse due to weak repulsive forces. Higher α (weaker attraction) requires shorter distances for amplification, but stronger repulsion increases the amplifiable range.

(e) Asymmetric elastic and magnetic forces in a symmetrical structure, and (f) its amplified motion as a function of distance when force anisotropy is controlled by γ, which is defined as the percentage difference between the two magnet-membrane pairs. The left pair consists of a membrane with modulus of (1-γ) E_0 and a magnet thickness of (1-γ) h_0, while the right pair uses a membrane with modulus of (1+γ) E_0 and a magnet with thickness of (1+γ) h_0. As γ increases, the stronger magnet is pulled more easily, enabling amplification over a larger range. However, larger γ causes weight imbalance, leading to uneven vibration motion between the magnets, even at the same distance from the electromagnet.

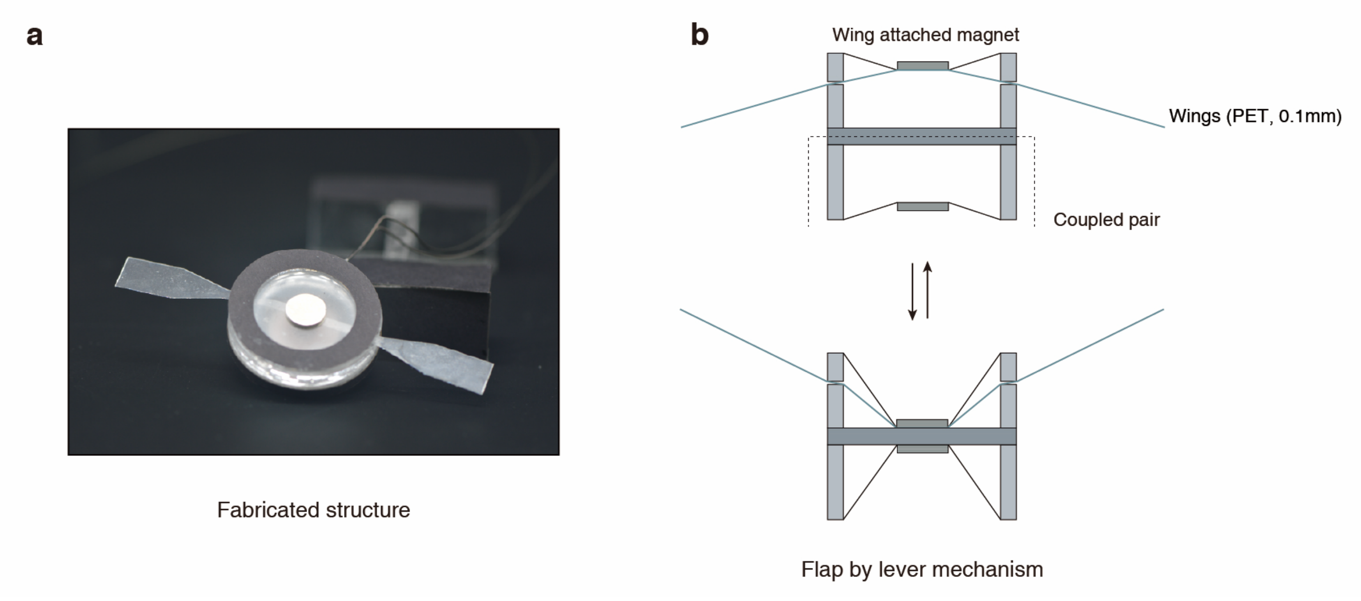

Supplementary Fig. 21. Structure and working mechanism for flapping wing demonstration.

(a) Photograph of C-EsMV system with PET film wings (0.1 mm) attached to a vibrating magnet. (b) The wing flaps through the lever mechanism as the magnet moves up and down.

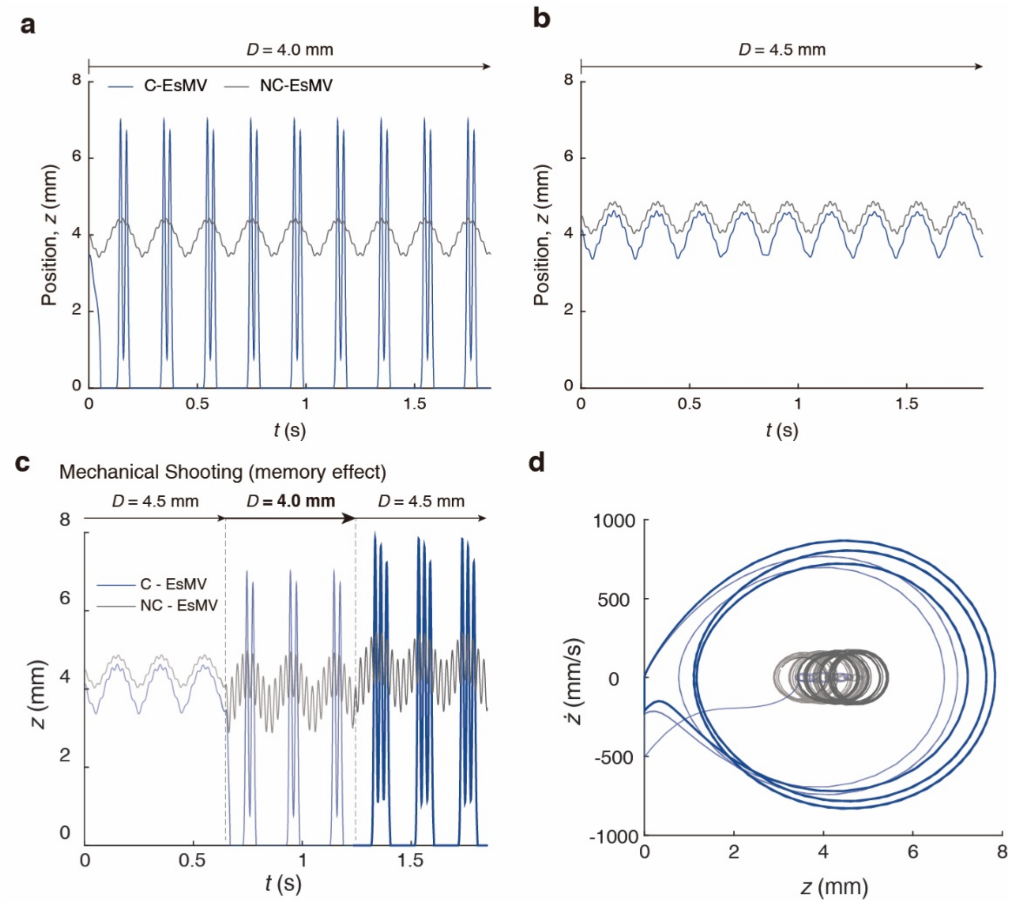

Supplementary Fig. 22. Numerical verification of mechanically triggered amplification.

Vibration motion of NC-EsMV and C-EsMV systems at an electromagnet-to-magnet distance of (a) D = 4.0 mm and (b) D = 4.5 mm, both under an input current of 2.0 A. (c) Analogous to the electrical shooting observed in Supplementary Fig. 13, mechanical triggering (D = 4.5 mm → 4.0 mm → 4.5 mm) also sustains amplified vibration in C-EsMV under constant input current, even after the trigger is removed. (d) Phase portraits of each system. The C-EsMV exhibits a larger-amplitude limit cycle (bold line) at the same input current and D, indicating greater vibration velocity and displacement compared with NC-EsMV.

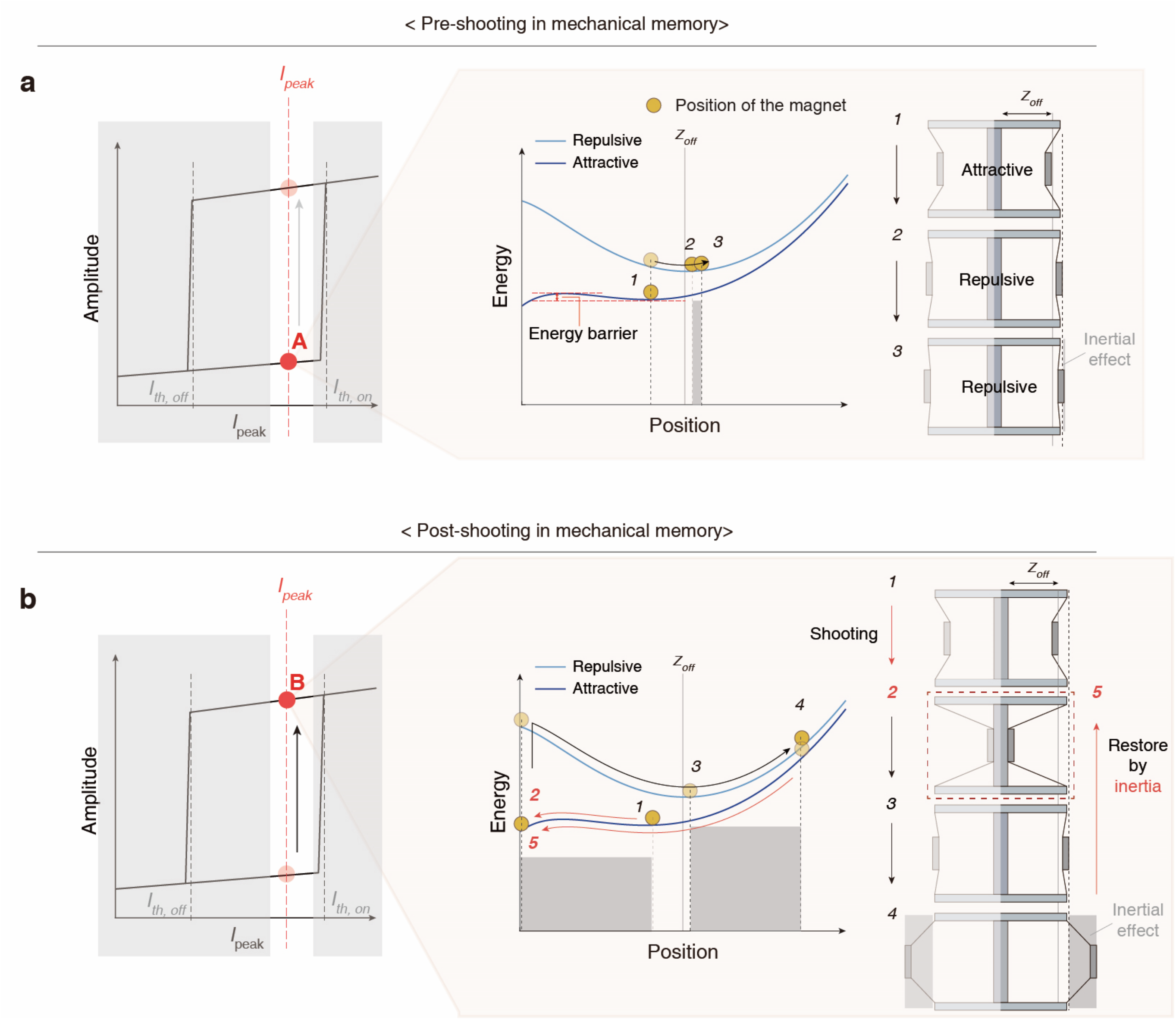

Supplementary Fig. 23. Mechanism of vibrational hysteretic behavior in mechanical memory.

Under identical input current I_{peak}, inertia allows two distinct vibration responses: (a) Pre-shooting (weakened state) and (b) post-shooting (amplified state). Without shooting, the magnet oscillates with small displacement near z_{off} (positions 1 – 3) unable to overcome the potential barrier imposed by magnetic attraction. As a result, the system remains in a low-amplitude vibration regime (Point A). By contrast, a brief perturbation (shooting)—which can be electrical, magnetic, or mechanical—pushes the magnet across the barrier to the bottom surface ($z = 0$, position "2"), storing additional elastic energy in the membrane. During the subsequent repulsive phase, this stored energy is released as a large overshoot ("3 → 4"), and the magnet returns with sufficient inertia to repeatedly cross the barrier ("4 → 1 → 5 (= 2)"). The residual kinetic energy from each cycle sustains these large-amplitude oscillations even after the external trigger is removed, corresponding to Point B on the amplitude–I_{peak} curve. If this residual energy is intentionally dissipated (see Supplementary Video 7), the system reverts to the weakened state.

Potential energy-position graphs are based on Supplementary Fig. 9e.

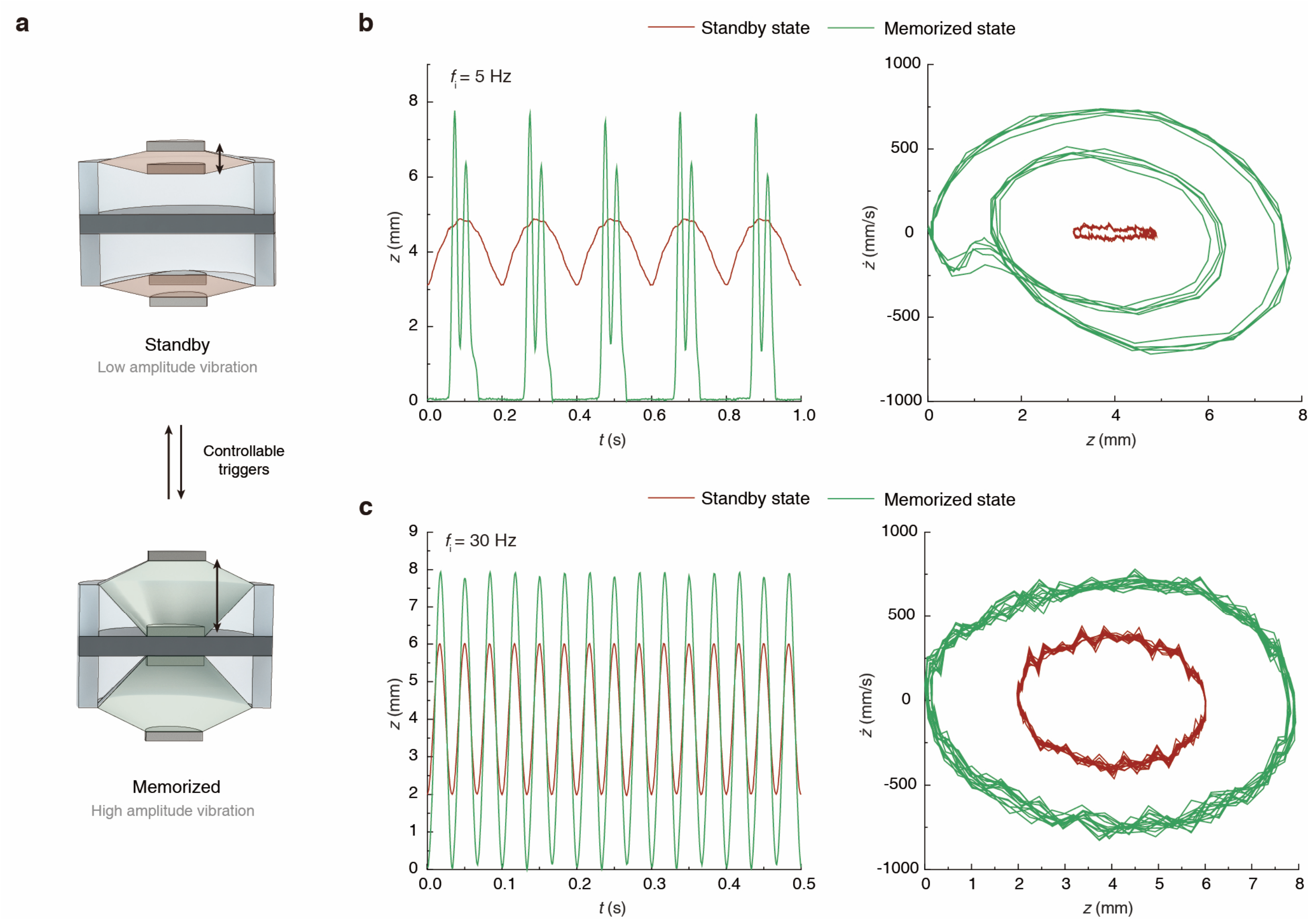

Supplementary Fig. 24. Experimental dynamic vibration response of the mechanical memory in standby and memorized states.

(a) Conceptual illustration of the mechanical memory. (b) Time-dependent vibration response and corresponding phase portrait at 5 Hz, comparing the standby (low-amplitude) and memorized (amplified) states. (c) Dynamic vibration response and phase portrait near resonance (30 Hz).

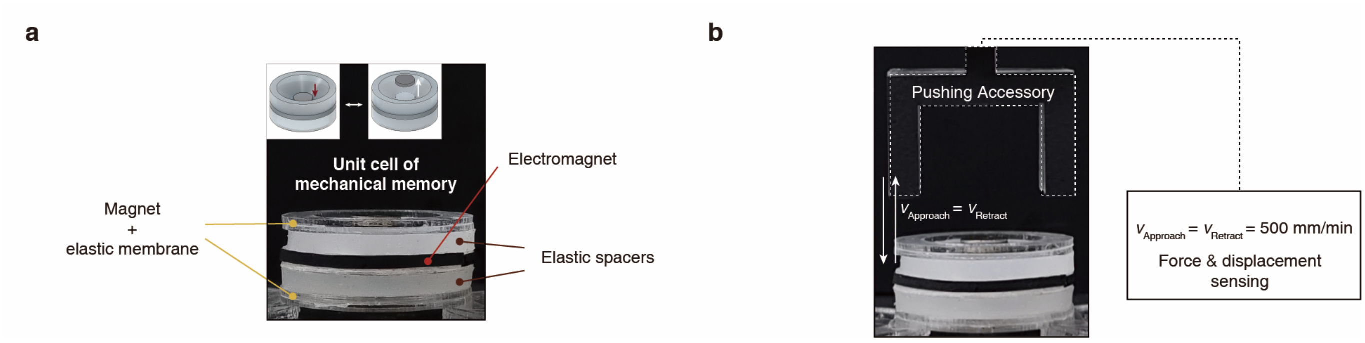

Supplementary Fig. 25. Experimental setup for mechanical memory and its operating modes.

(a) Basic unit cell of the mechanical memory system. (b) The unit cell is triggered using a push accessory at a speed of 500 mm/min, with approach and retract speeds indicated.

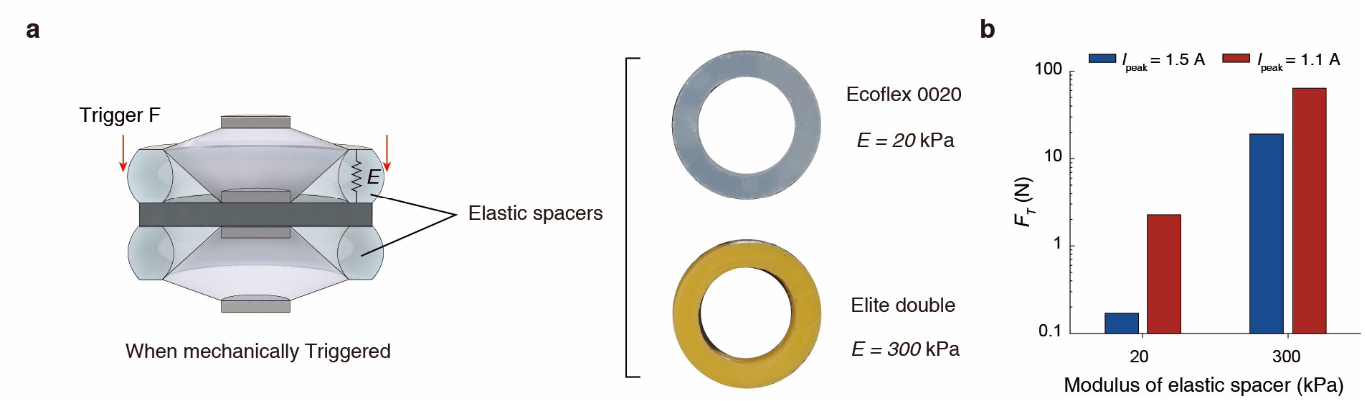

Supplementary Fig. 26. Trigger force characterization with elastic spacers of different elastic moduli.

(a) Schematic illustration of the elastic spacer setup, showing two spacers with different moduli, $E = 20$ kPa and $E = 300$ kPa. (b) Trigger force required for amplified vibration in each spacer. When the input current is close to the threshold ($I \approx I_{th}$, $I_{peak} = 1.5$ A), even small forces can surpass the threshold, resulting in low trigger force. As the current deviates from I_{th} ($I_{peak} = 1.1$ A), greater force is required to achieve amplification. Experiments were conducted under *R3* conditions, where amplification is sustained after the trigger in both cases.

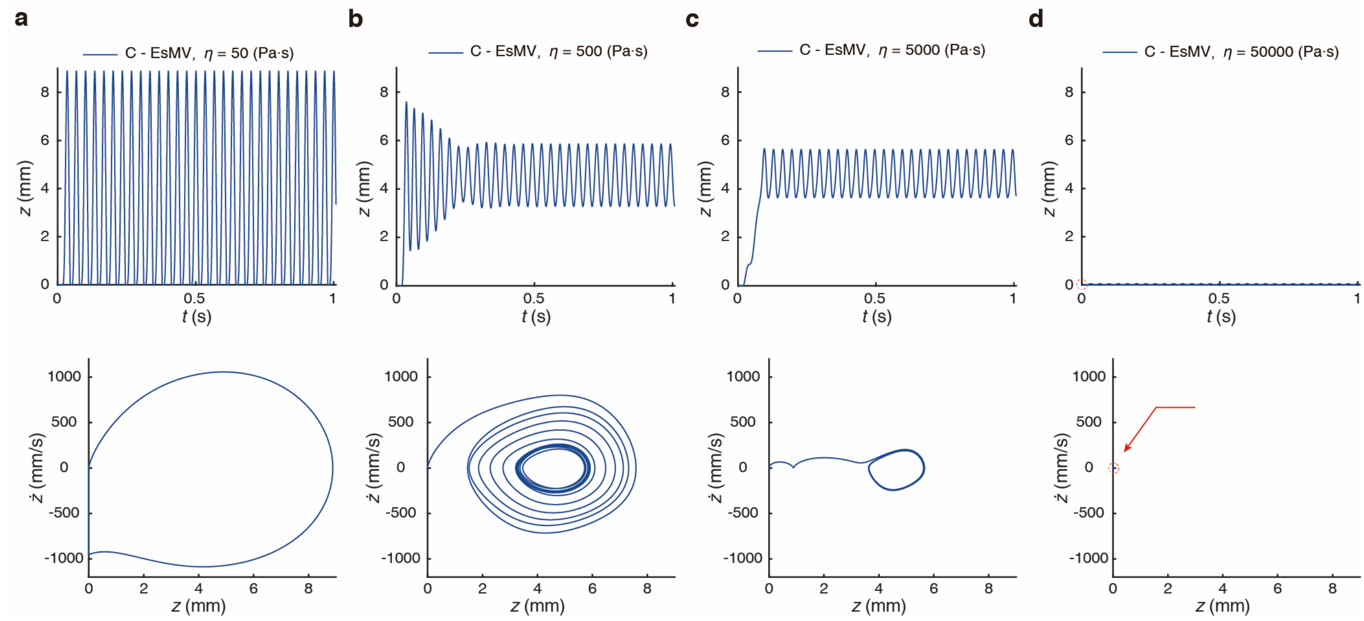

Supplementary Fig. 27. Effect of membrane damping on mechanical memory. (a) η = 50 (b) η = 500 (c) η = 5000 (d) η = 50000 Pa·s.

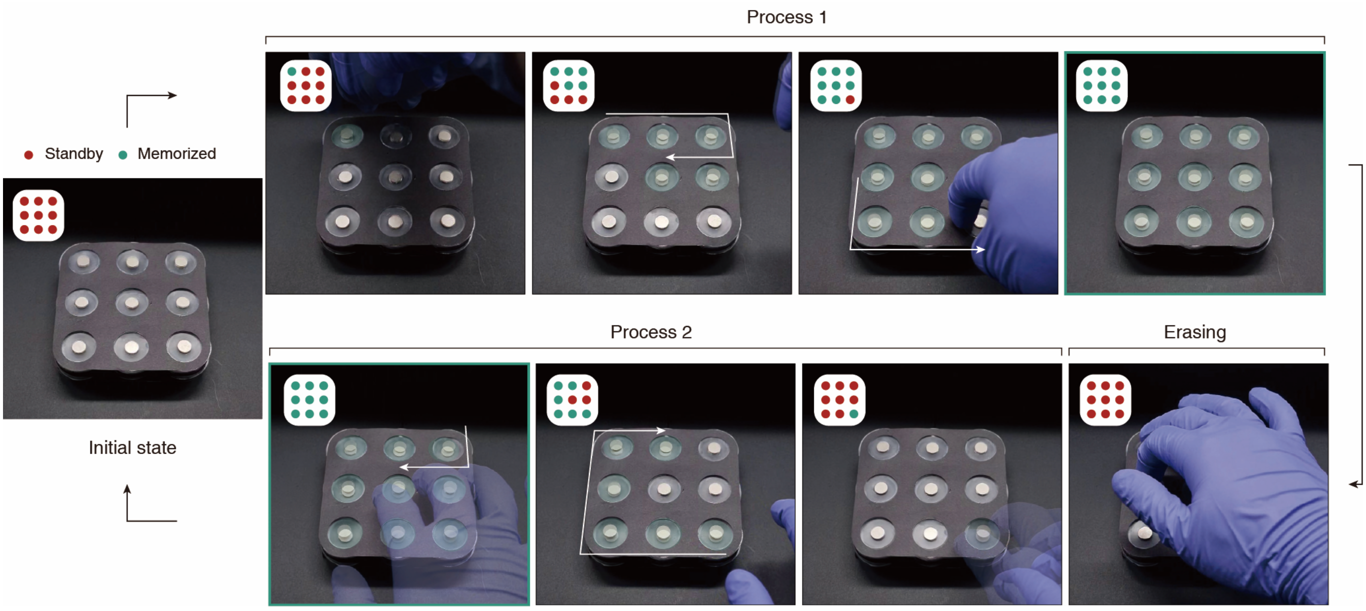

Supplementary Fig. 28. The entire process of non-volatile mechanical memorization.

When a trigger displacement is applied by pressing the spacer from the standby state, the system traces and stores the mechanical trigger. By placing a hand near the vibrating magnet, the energy is dissipated, erasing the memory. The system can then re-memorize the trigger, regardless of the previous path.

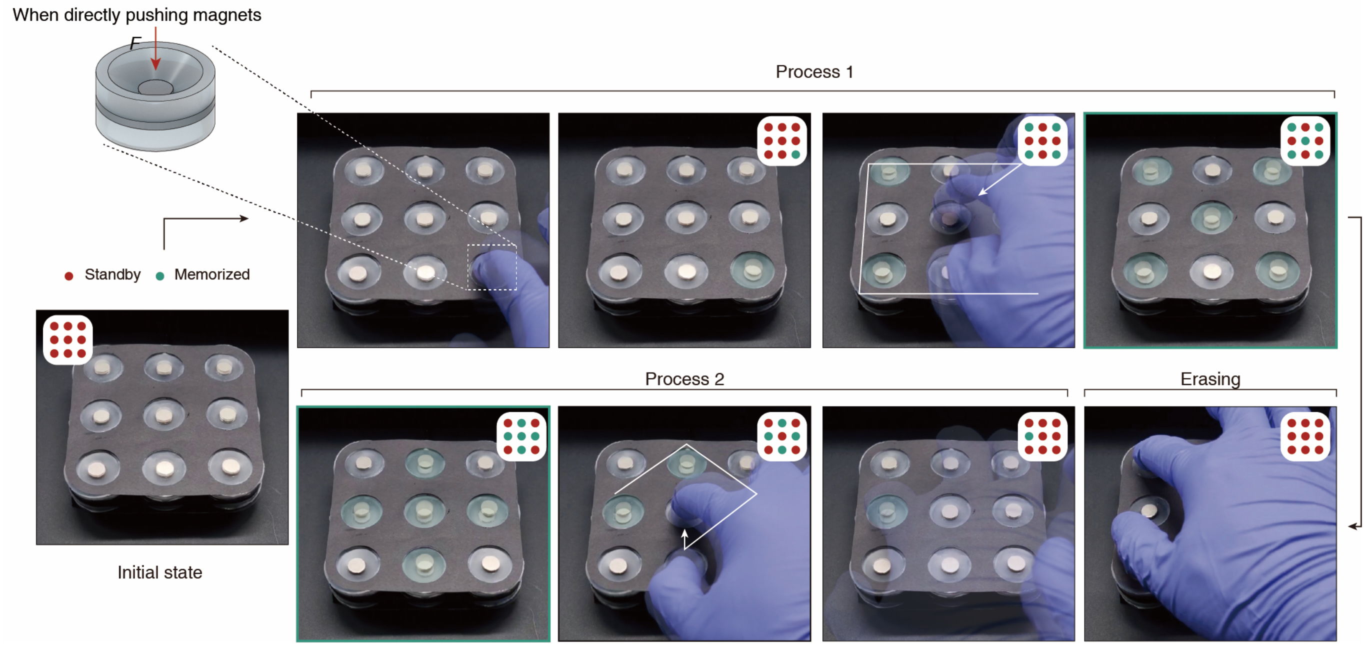

Supplementary Fig. 29. Non-volatile mechanical memorization by directly pressing the magnet.

Pressing the magnet attached to the membrane, instead of the spacer, raises the maximum elastic potential energy. This creates a similar effect to mechanical shooting, enabling amplified vibrations and non-volatile mechanical memorization.

Supplementary references.

1. Ogden, R. W. Non-linear elastic deformations. *Courier Corporation* (1997).
2. Thomson, W. IV. On the elasticity and viscosity of metals. *Proc. R. Soc. Lond.* **14**, 289-297 (1865).
3. Sosa, V. Alternative method to calculate the magnetic field of permanent magnets with azimuthal symmetry. *Revista mexicana de física E*, **59**, 8-17 (2013).
4. Pollack, G. L. & Stump, D. R. *Electromagnetism*, (Addison-Wesley, Boston, 2001).
