# Supplementary material for: Elasto-magnetic instabilities for amplified actuation and mechanical memory
Source: Nat Commun. 2026 Jan 10;17:1511. doi: 10.1038/s41467-025-68225-y (PMC12890967; doi:10.1038/s41467-025-68225-y)
Supplement: Supplementary file 2 — Description of Additional Supplementary Files [file 41467_2025_68225_MOESM2_ESM.pdf]

Description of Additional Supplementary Files

Supplementary Video 1. Amplified elasto-magnetic vibrations via magnetic coupling

Comparison of non-coupled (NC-EsMV) and coupled (C-EsMV) vibrations at 5 Hz and 30 Hz. At near-resonant frequencies, magnetic coupling further enhances vibration amplitude due to inertial effects.

Supplementary Video 2. Nonlinear response with vibrational hysteresis

The system exhibits stepwise amplification and hysteresis as the peak input current is gradually increased and decreased at both 5 Hz and 30 Hz. Retained vibrations below the activation threshold highlight inertia-driven hysteresis behavior.

Supplementary Video 3. Non-contact, non-volatile mechanical memory

Demonstration of memory activation in a 3×3 array via non-contact magnetic triggers. The system transitions from a weakened to an amplified state under constant sub-threshold AC input, maintaining the memory state without further input. Operated at 30 Hz; visual timing may be affected by video frame rate.

Supplementary Video 4. Distinct vibration states: *Collapsed, Amplified, Weakened*

Visualization of three operational regimes controlled by the initial position D. At 30 Hz, resonance enables sustained amplification over a broader range of distances.

Supplementary Video 5. Amplification of wing-like flapping motion

A magnet-attached wing structure demonstrates limited actuation under NC-EsMV. Coupling dramatically increases flapping amplitude, showcasing enhanced motion transfer.

Supplementary Video 6. Tunable mechanical memory regimes (R1-R4)

Demonstration of four memory regimes by varying peak input current. Each regime corresponds to a different memory behavior: non-memorizable, volatile, non-volatile, or persistently amplified.

Supplementary Video 7. Volatile and non-volatile mechanical memory array

A 3×3 mechanical memory array encodes trigger events across R2 and R3 regimes, distinguishing between volatile and non-volatile responses. Operated at 30 Hz; visual timing may be affected by video frame rate.
